# *Limosilactobacillus reuteri* in immunomodulation: molecular mechanisms and potential applications

**DOI:** 10.3389/fimmu.2023.1228754

**Published:** 2023-08-09

**Authors:** Zichen Luo, Ailing Chen, Anni Xie, Xueying Liu, Shanyu Jiang, Renqiang Yu

**Affiliations:** ^1^ Department of Neonatology, Women’s Hospital of Jiangnan University, Wuxi Maternity and Child Health Care Hospital, Wuxi, China; ^2^ Research Institute for Reproductive Health and Genetic Diseases, Women’s Hospital of Jiangnan University, Wuxi Maternity and Child Health Care Hospital, Wuxi, China

**Keywords:** *Limosilactobacillus reuteri*, immune system disease, atopic dermatitis (AD), asthma, systemic lupus erythematosus (SLE), rheumatoid arthritis, multiple sclerosis, necrotizing enterocolitis (NEC)

## Abstract

Frequent use of hormones and drugs may be associated with side-effects. Recent studies have shown that probiotics have effects on the prevention and treatment of immune-related diseases. *Limosilactobacillus reuteri* (*L. reuteri*) had regulatory effects on intestinal microbiota, host epithelial cells, immune cells, cytokines, antibodies (Ab), toll-like receptors (TLRs), tryptophan (Try) metabolism, antioxidant enzymes, and expression of related genes, and exhibits antibacterial and anti-inflammatory effects, leading to alleviation of disease symptoms. Although the specific composition of the cell-free supernatant (CFS) of *L. reuteri* has not been clarified, its efficacy in animal models has drawn increased attention to its potential use. This review summarizes the effects of *L. reuteri* on intestinal flora and immune regulation, and discusses the feasibility of its application in atopic dermatitis (AD), asthma, necrotizing enterocolitis (NEC), systemic lupus erythematosus (SLE), rheumatoid arthritis (RA), and multiple sclerosis (MS), and provides insights for the prevention and treatment of immune-related diseases.

## 1 Introduction

In recent years, with the development of the concepts of gut-lung axis, gut-brain axis, gut-liver axis, and gut-skin axis, increased attention has been paid to the impact of intestinal flora on health. In particular, the discovery that probiotics can reshape gut flora (1) has triggered a wave of research focused on the gut microbiome. Probiotics colonize and regulate the unbalanced microflora of the host, directly acting on host epithelial and immune cells and regulating the epithelial or immune cells of specific tissues of the host through their metabolites *in situ* or by entering the circulation and remodeling the microenvironment of the lesion site (2). Unlike drugs, probiotics are associated with less side-effects, such as intestinal ecological disorder and immune dysfunction (3). Various forms of probiotic preparations are available. The employment of heat-killed probiotics, cell-free supernatant (CFS) of probiotics and specific components of purification, or genetic engineering editing of probiotics to obtain certain specific functions and avoid side-effects is an emerging trend in this field (4).

Lactobacillus is one of the earliest discovered probiotics (5) and is most commonly used in foods and dietary supplements (6). It can be isolated from a wide range of sources, including human or animal milk, feces, and fermented foods (7–9). *Lactobacillus acidophilus*, *Lactobacillus rhamnosus* (*L. rhamnosus)*, and *Limosilactobacillus reuteri* (*L. reuteri*) are currently the most widely used probiotics (10–12). Probiotics have a wide range of functions, including resistance to pathogenic microorganisms, regulation of immunity, regulation of metabolism such as cholesterol and sugar metabolism, antioxidant, and antitumor effects. Notably, the probiotic-related protective mechanisms of lactobacilli on the host are strain-dependent (13).

*L. reuteri* is one of the most studied strains of the *Lactobacillus* genus. Its morphology and growth are affected by the host genotype and environmental factors, such as temperature, pH, oxygen concentration, and host dietary components. Genetic and environmental differences lead to phenotypic heterogeneity of *L. reuteri* strains (14, 15). *L. reuteri* may play a regulatory role in several systemic diseases through a very intricate immunoregulatory mechanism. First, it colonizes and survives in the gastrointestinal tract by utilizing its acid-base resistant and adhesion properties. Subsequently, it interacts with host intestinal epithelial cells (IECs), by regulating the intestinal flora, to enhance intestinal mucosal barrier, regulate immune cells, inflammatory factors, chemokines, and antibodies, produce indole derivative from tryptophan, secrete exopolysaccharide (EPS) and other bioactive factors, enhance tight junctions (TJs), regulate gene expression, improve antioxidant activity, and further regulate the immune system of the host. As such, *L. reuteri* has the potential to be used as a new therapeutic or adjunctive therapy in atopic or autoimmune diseases. Previous reviews have focused on the use of *L. reuteri* in gastrointestinal diseases such as colic and diarrhea. Based on its potential role in immune regulation, this review summarizes the immunomodulatory molecular mechanisms of different strains of *L. reuteri*. We searched for studies on the application of *L. reuteri* in disease and found that it was applied to diseases or disease models in multiple systems, with digestive and immune-related diseases being the most common. In these two categories of diseases, the existing reviews and meta-analyses focused on a single *L. reuteri* strain or a single digestive system disease, and no review summarized the application of different strains of *L. reuteri* in immune-related diseases. This review summarized the studies on the application of *L. reuteri* in immune-related diseases. We explored the application value of *L. reuteri* in these immune-related diseases such as atopic dermatitis, asthma, necrotizing enterocolitis, systemic lupus erythematosus, rheumatoid arthritis, and multiple sclerosis.

## 2 Immune regulation mechanism

For probiotics to exert their beneficial effects on the host organism, some basic characteristics should be fulfilled. First, they need to be resistant to acid and bile salts to survive the passage through the gastrointestinal tract. Second, they should be able to adhere to mucus and IECs to colonize the host. Only after these conditions are met can probiotics exert their anti-infection, anti-inflammatory, and antioxidant properties in the host (16).

### 2.1 Changes in microbial structure, metabolites, and expression of functional genes

#### 2.1.1 Regulation of microorganisms

**Figure 1:**
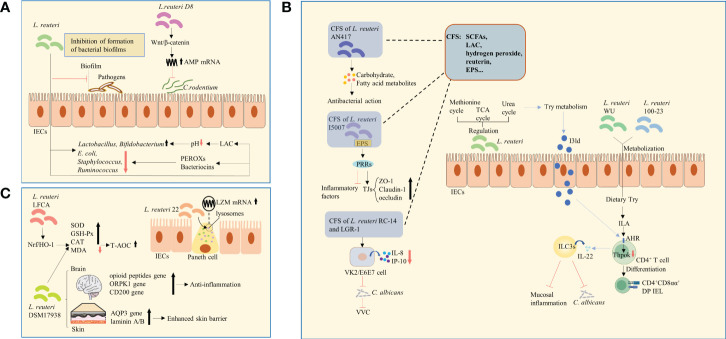
Changes in microbial structure, metabolites, and expression of functional genes. **(A)** *L. reuteri* regulated the relative abundance of microorganisms. The antibacterial effect of *L. reuteri* is achieved through the production of LAC, which regulates the local pH and facilitates the growth of beneficial bacteria, or through the production of PEROXs and bacteriocins*. L. reuteri* activated the Wnt/β-catenin pathway, leading to increased expression of AMPs, thus inhibiting the colonization of *C. rodentium. E.coli, Escherichia* coli; LAC, lactic acid; PEROXs, peroxides; AMPs, antimicrobial peptides. **(B)** *L. reuteri* increased the levels of amino acid metabolites by regulating the urea, TCA, and methionine cycles, thus enhancing Try metabolism and in turn the anti-inflammatory ability. *L. reuteri* metabolized dietary Try to produce IAld, which acts as a ligand for the activation of the AHR on the surface of CD4^+^ T-cells, and induces ILC3s to produce IL-22. This process enhanced host resistance to *C. albicans* and protected against mucosal inflammation. Similarly, through the activation of Try metabolism and production of ILA that activate AHR and downregulate Thpok, *L. reuteri* promoted the differentiation of CD4^+^ T-cells into CD4^+^CD8αα^+^ DP IELs. The CSF of *L. reuteri* includes some of its metabolites and secreted bioactive factors, such as SCFAs, organic acids (such as LAC), hydrogen peroxide, bacteriocin compounds (such as reuterin), and EPS. The carbohydrate and fatty acid metabolites in CFS of *L. reuturi* AN417 has antibacterial action against oral pathogens. Binding of EPS of *L. reuteri* I5007 to PRRs was suggested to downregulate inflammatory factors and upregulate claudin-1, occludin, and ZO-1, thus enhancing intestinal barrier function. The CFS of *L. reuteri* RC-14 combined with that of LGR-1 inhibited the secretion of IL-8 and IP-10. It was shown to inhibit the colonization and growth of C. albicans and occurrence of VVC. TCA, tricarboxylic acid; Try, tryptophan; IAld, indole-3-aldehyde; AHR, aryl hydrocarbon receptor; ILC3s, group 3 innate lymphoid cells; IL-22, interleukin-22; *C. albicans, Candida albicans*; ILA, indole lactic acid; DP IELs, double-positive intraepithelial lymphocytes; CFS, Cell-free supernatant; EPS, exopolysaccharide; PRRs, pattern recognition receptors; ZO-1, zonula occludens 1; *LGR-1*, *Lacticaseibacillus rhamnosus GR-1*; IP-10, interferon-inducible protein-10; VVC, vulvovaginal candidiasis; SCFAs, short-chain fatty acids; LAC, lactic acid. **(C)** *L. reuteri* 22 promoted the mRNA expression of LZM and enhanced congenital immunity of intestinal mucosa. *L. reuteri* DSM 17938 increased the expression of skin epidermal AQP3 and lamininA/B, which strengthens skin barrier function. It also upregulated the expression of genes of opioid peptide, OPRK1, and CD200, which are related to stress and pain in the brain and are involved in anti-inflammatory signaling pathways. *L. reuteri* -LFCA improved oxidative stress-related indicators by activating Nrf2/HO-1 signaling pathway. The level of MDA was reduced, whereas those of enzymes, such as SOD, GSH-Px, and catalase were increased. *L. reuteri* DSM 17938 had similar effects. LZM, lysozyme; AQP3, aquaporin 3; kappa-opioid receptor 1, OPRK1; Nrf2/HO-1, nuclear factor E2-related factor 2/Heme oxygenase1; MDA, malondialdehyde; SOD, superoxide dismutase; GSH-Px, glutathione peroxidase.

The homeostasis of intestinal microorganisms is essential for effective host intestinal barrier function and normal immune responses (17). An imbalance in intestinal microecology decreases the immune tolerance of the host to allergens and causes autoimmune reactions (18). For example, *L. reuteri* is known to adhere to and aggregate mucus and IECs, hindering the interaction of pathogens with the host, thus constituting a defensive barrier against invading pathogens (19). *L. reuteri* has also been reported to enrich intestinal microbial diversity and regulate the relative abundance of adminis, mainly manifesting as an increase in the numbers of beneficial genera and decrease in those of harmful genera. For example, studies have shown that following the administration of *L. reuteri*, the numbers of *Lactobacillus* and *Bifidobacterium* were increased (20, 21), whereas those of *Escherichia coli (E.coli)*, *Staphylococcus*, and *Ruminococcus* were decreased (22, 23), thus balancing intestinal microecology (**Figure 1A**). However, no consensus has been reached regarding its influence on microbial composition by heredity, environment, and strain specificity. The antibacterial effect of *L. reuteri* is achieved through the production of organic acids, which regulate the local pH and facilitate the growth of beneficial bacteria (24), or through the production of peroxides(PEROXs) and reuterin that inhibit the growth of pathogens (25) (**Figure 1A**). Among these antimicrobial metabolites, reuterin is of the most significance. It metabolizes glycerol to hydroxypropionaldehyde (3-HPA) (26). It’s the chemical nature of reuterin. This process is mediated by glycerol dehydratase and assisted by coenzyme B12 (27, 28). 3-HPA is further converted into acrolein, which exerts cytotoxic effects and inhibits the growth of gram-negative bacilli. This may be the mechanism by which reuterin exerts its antibacterial effect (29, 30). *L. reuteri* has also been shown to inhibit the formation of bacterial biofilms (**Figure 1A**). In addition, *L. reuteri* inhibited the gene expression of invading bacteria, thus limiting their growth and virulence (25). In particular, *L. reuteri* activated the Wnt/β-catenin pathway, leading to increased expression of antimicrobial peptides (AMPs), thus inhibiting the colonization of *C. rodentium* (31) (**Figure 1A**). However, some studies have reported that *L. reuteri* had no effect on the abundance of *E. coli* and Group B *Streptococcus* (GBS) strains (32, 33).

#### 2.1.2 Metabolites

Tryptophan catabolites: Normal tryptophan (Try) metabolism is an important process in maintaining intestinal mucosal homeostasis. Notably, *L. reuteri* increased the levels of amino acid metabolites by regulating the urea, tricarboxylic acid, and methionine cycles, thus enhancing Try metabolism and in turn the anti-inflammatory ability in mice (23). Studies have shown that *L. reuturi* metabolizes dietary Try to produce indole-3-aldehyde (IAld), which acts as a ligand for the activation of the aryl hydrocarbon receptor (AHR) on the surface of CD4^+^ T-cells (34), and induces group 3 innate lymphoid cells (ILC3s) to produce interleukin-22 (IL-22) (35). This process enhanced host resistance to *Candida albicans* (*C. albicans*) and protected against mucosal inflammation (34) (**Figure 1B**). Similarly, through the activation of Try metabolism and production of indoles that activate AHR and downregulate the transcription factor Thpok, *L. reuteri* promoted the differentiation of CD4^+^ T-cells into CD4^+^CD8αα^+^ double-positive intraepithelial lymphocytes (DP IELs) (36) (**Figure 1B**). Therefore, promoting Try catabolism might be one of the mechanisms by which *L.reuturi* exerts its anti-inflammatory and anti-infection properties.

Cell-free supernatant (CFS): The *L. reuturi* AN417 strain has no antibacterial action against oral pathogens, whereas its carbohydrate and fatty acid metabolites in CFS do (37) (**Figure 1B**). Similarly, *L. reuturi* I5007 exerts regulatory effects on inflammatory factors and TJs (38). Binding of EPS, a possible CFS component, to pattern recognition receptors (PRRs) (39), was suggested to downregulate inflammatory factors and upregulate TJs, such as claudin-1, occludin, and zonula occludens 1 (ZO-1), thus enhancing intestinal barrier function (38) (**Figure 1B**). Of note, the CFS of *L.reuteri* RC-14 alone or combined with *Lacticaseibacillus rhamnosus GR-1 (LGR-1)* was not effective against vulvovaginal candidiasis (VVC), whereas their combined CFS inhibited the secretion of IL-8 and chemotactic factor interferon-inducible protein-10 (IP-10) from human vaginal epithelial VK2/E6E7 cells. Eventually, it was shown to inhibit the colonization and growth of *C. albicans* and occurrence of VVC (40) (**Figure 1B**). The CSF of *L. reuteri* includes some of its metabolites and secreted bioactive factors, such as short-chain fatty acids (SCFAs), organic acids (such as LAC), hydrogen peroxide, bacteriocin compounds (such as reuterin), and EPS (**Figure 1B**). Several recent studies have demonstrated the effectiveness of CFS. The specific active components and their mechanisms of action can be further explored in the future, potentially enabling major discoveries in the field of microbiome research.

#### 2.1.3 Regulation of gene expression

**Figure 2:**
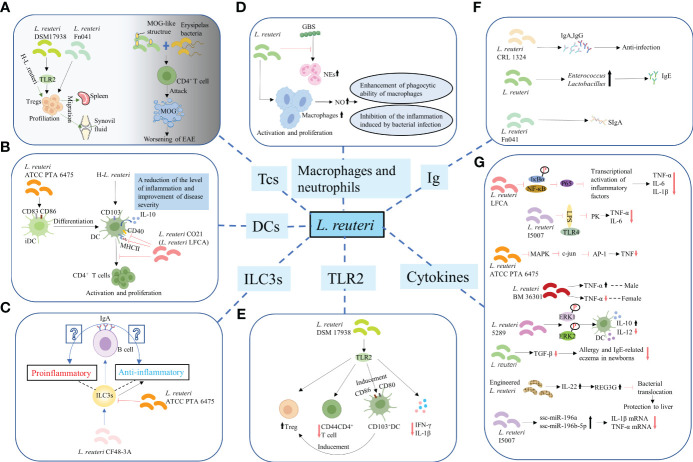
Regulation of the differentiation and function of immune cells. **(A)** T-cells. *L. reuteri* 17938 and *L. reuteri* Fn041 stimulated the production of Foxp3^+^ Treg cells. Similarly, *h-L.reuteri* increased the numbers of Treg cells in the spleen and joint drainage lymph nodes. *L. reuteri* has a structure similar to that of the mouse MOG. It cooperated with erysipelas bacteria to activate CD4^+^ T-cells, which have the ability to attack MOG, leading to worsening of EAE. h-*L. reuteri*, heat-killed *L. reuteri*; MOG, myelin oligodendrocyte glycoprotein; EAE, experimental autoimmune encephalomyelitis. **(B)** Dendritic cells. *L. reuturi* ATTC PTA 6475 and its secreted factors induced the expression of CD83 and CD86 on the surface of iDCs and promoted the production of IL-10 by DCs. *L. reuteri* CO21 inhibited the expression of CD40 and MHCII on the surface of DCs and the activation and proliferation of CD4 ^+^ T-cells induced by DCs, thus achieving an anti-inflammatory effect. *H-L.reuteri* increased the numbers of CD103 ^+^ DCs, resulting in a reduction of the level of inflammation and improvement of disease severity. iDCs, immature dendritic cells; IL-10, interleukin-10; DCs, dendritic cells. **(C)** ILC3s. *L. reuteri* ATCC PTA 6475 reduced the number of ILC3s. *L. reuteri* CF48-3A promoted the production of ILC3s and induced the differentiation of IgA^+^ B–cells, leading to the generation of IgA. However, whether the IgA play a protective role against infection and enhances mucosal immunity or induces autoimmune effects remains unclear. ILC3s, Group 3 innate lymphoid cells. **(D)** Macrophages and NEs. *L. reuteri* increased production of NO by activated and increased numbers of macrophages, enhancing their phagocytotic ability. Meanwhile, *L. reut*eri inhibited the inflammation induced by bacterial infection by regulating the levels of NO. However, *L. reuteri* reduced the GBS-induced proliferation of NEs. NEs, neutrophils; NO, nitric oxide; GBS, Group B Streptococcus. **(E)** TLR2. *L. reuteri* 17938 is a bacterium that following recognition by TLR2 mediates anti-inflammatory immune response in the following ways: First, it led to an increase in the number of Foxp3^+^ Treg cells and a decrease in that of CD4^+^CD44^+^ Teffs. Second, it induced the generation and activation of CD103^+^ DCs, which were characterized by the expression of co-stimulative markers CD80 and CD86. This DC-induced production of Foxp3^+^ Treg cells is crucial for maintaining intestinal immune tolerance. Finally, activation of TLR2 mediated the decrease in the levels of IL-1β and IFN-γ.TLR2, Toll-like receptor 2; Teffs, effector T-cells; IFN-γ, interferon-γ. **(F)** Promotion of synthesis and secretion of Ig. *L. reuteri* FN041 increased the synthesis of sIgA. *L. reuteri* CRL1324 not only stimulated the production of IgA, but also that of IgG, which is related to anti-infection functions. An increase in the numbers of lactobacilli and enterococci caused by administration of mixed strains of *L. reuteri* were correlated with increased levels of IgE. Ig, Immunoglobulin; sIgA, secreted IgA. **(G)** Regulation of cytokines for improving intestinal mucosal barrier structure and permeability.

A study found that *L. reuteri* 22 greatly promoted the mRNA expression of lysozyme (LZM) and enhanced congenital immunity of intestinal mucosa (41) (**Figure 1C**). In addition, a number of differentially expressed microRNAs (miRNAs) were detected in newborn piglets that were orally administered *L. reuteri* I5007. These miRNAs were involved in the phosphatidylinositol-3-hydroxykinase (PI3K)-protein kinaseB (AKT) and mitogen-activated protein kinase (MAPK) pathways, playing an important role in the probiotic-host crosstalk. In addition, the expression of ssc-miR-196a/-196b-5p was significantly increased, downregulating the mRNA expression of IL-1β and TNF-α in IPEC-J2 cells (42) (**Figure 2G**). *L. reuteri* DSM 17938 was reported to significantly increase the expression of skin epidermal aquaporin 3 (AQP3) and lamininA/B, which strengthens skin barrier function (43) (**Figure 1C**), and upregulated the expression of genes of opioid peptide, kappa-opioid receptor 1 (OPRK1), and CD200, which are related to stress and pain in the brain and are involved in anti-inflammatory signaling pathways (44) (**Figure 1C**). *In vitro* experiments have shown that oral administration of *L. reuteri* -LFCA improved oxidative stress-related indicators by activating the nuclear factor E2-related factor 2 (Nrf2)/Heme oxygenase1 (HO-1) signaling pathway (45, 46). Moreover, the level of malondialdehyde (MDA) was reduced, whereas those of enzymes, such as superoxide dismutase (SOD), glutathione peroxidase (GSH-Px), and catalase were increased (46) (**Figure 1C**), thus enhancing the antioxidant capacity of cells. Similar effects of *L. reuteri* DSM 17938 (**Figure 1C**)—used as a new treatment for reducing intestinal inflammation and repairing intestinal damage—have been reported (47).

### 2.2 Protection of the integrity of intestinal mucosal barrier

**Figure 3:**
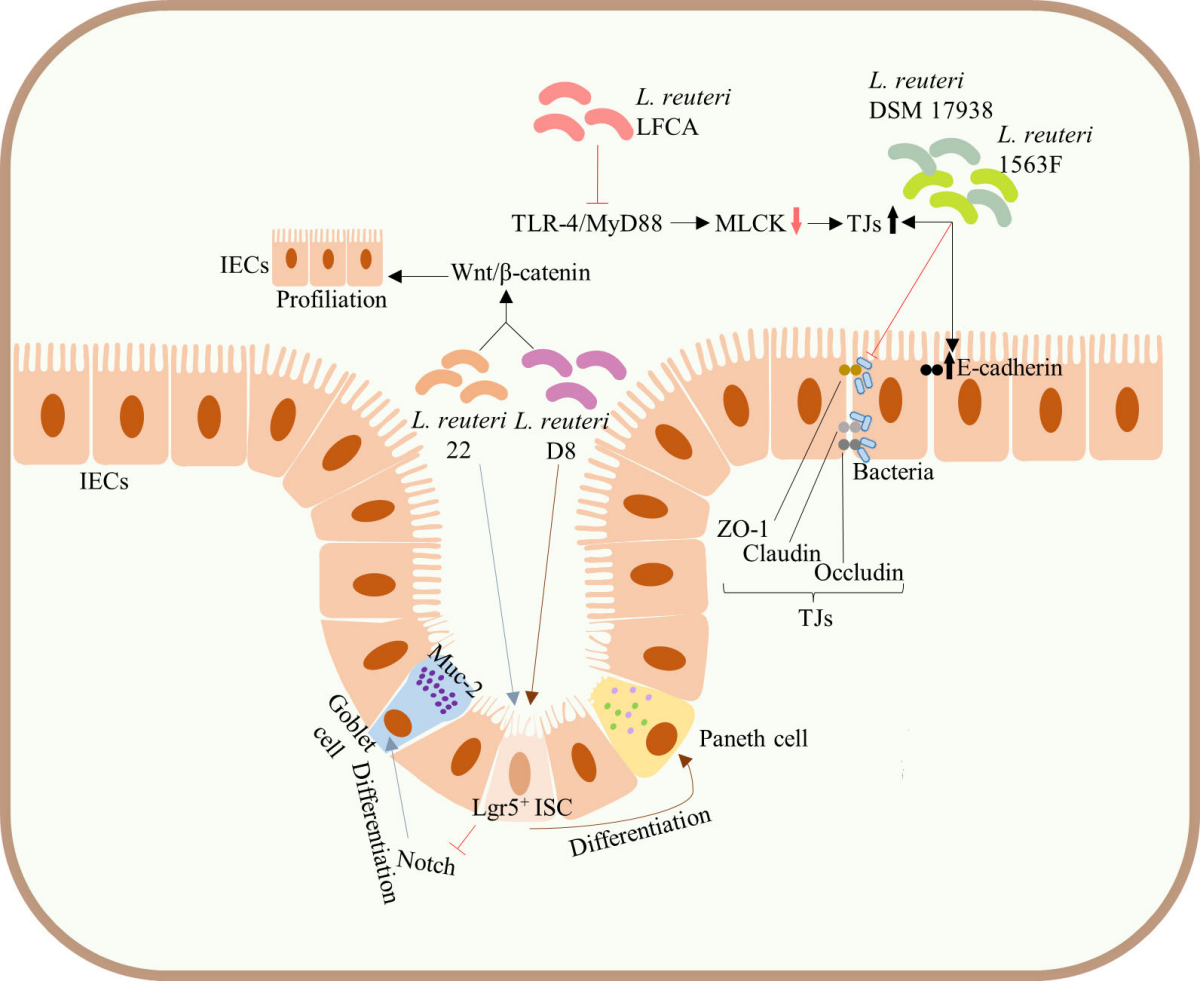
Protection of the integrity of intestinal mucosal barrier. Administration of both *L. reuteri* D8 and *L. reuteri* 22 increased the number of ISCs, Lgr5^+^ cells, and promoted the proliferation of IECs by activating the Wnt/β-catenin pathway. *L. reuteri* 22 inhibited Notch signaling pathway and induced the differentiation of ISCs into Muc-2-highly expressing goblet cells, whereas *L. reuteri* D8 induced the differentiation of ISCs into Paneth cells. Both *L. reuteri* DSM17938 and 1563F upregulated the expression of E-cadherin and TJs in infected IECs and competitively inhibited bacterial binding to TJs, thus inhibiting the increased infection-induced intestinal permeability and protecting intestinal barrier function. Upregulation of the expression of TJs in intestinal epithelium can be achieved by inhibiting the TLR4/MyD88 signal transduction pathway and downregulating the MLCK pathway. ISCs, intestinal stem cells; mucin-2, Muc-2; IECs, intestinal epithelial cells; TJs, tight junctions; TLR4, Toll-like receptor 4; MyD88, myeloid differentiation factor 88; MLCK, myosin light chain kinase.

The integrated intestinal barrier structure and its normal function constitute a meaningful immune defense barrier against pathogenic microbes. In addition to the above pathways, *L. reuteri* can also directly enhance intestinal barrier function by acting on intestinal stem cells (ISCs) and TJs of IECs. *L. reuteri* is known to promote the development and differentiation of IECs, maintaining the integrity of intestinal mucosa. For example, administration of both *L. reuteri* D8 and *L. reuteri* 22 increased the number of ISCs, Lgr5^+^ cells, and promoted the proliferation of IECs by activating the Wnt/β-catenin pathway (31, 41). Of note, *L. reuteri* 22 inhibited Notch signaling pathway and induced the differentiation of ISCs into mucin-2 (Muc-2)-highly expressing goblet cells (41), whereas *L. reuteri* D8 induced the differentiation of ISCs into Paneth cells (31) (**Figure 3**), both of which are significant components of the intestinal barrier. Similarly, *L. reuteri* I5007 upregulated the expression of TJs, such as occludin, claudin, and ZO-1 of small IECs in piglets, thereby enhancing the intestinal mucosal barrier (38) (**Figure 1B**). Both *L. reuteri* DSM17938 and 1563F upregulated the expression of E-cadherin and TJs in infected IECs and competitively inhibited bacterial binding to TJs, thus inhibiting the increased infection-induced intestinal permeability and protecting intestinal barrier function (48) (**Figure 3**). Meanwhile, use of peptidoglycans on skin epidermis activated TLR2, resulting in the increased expression of TJs and enhanced skin barrier function (38, 39). However, whether peptidoglycans of probiotics can enhance the intestinal mucosal barrier through a similar mechanism of action remains to be clarified. Current animal studies have shown that the upregulation of the expression of TJs in intestinal epithelium can be achieved by inhibiting the TLR4/myeloid differentiation factor 88 (MyD88) signal transduction pathway and downregulating the myosin light chain kinase (MLCK) pathway (46) (**Figure 3**).

### 2.3 Regulation of the differentiation and function of immune cells

#### 2.3.1 T-cells

Various studies have reported that *L. reuteri* not only promoted the increase of DPIELs in mice (36) but also induced the generation of regulatory T (Treg)-cells. For example, *L. reuteri* 17938 and *L. reuteri* Fn041 stimulated the production of Foxp3^+^ Treg cells in the intestinal mucosa (49–51) (**Figure 2A**), the reduction of crying in infants with colic was thought to be the therapeutic effect of *L. reuteri* 17938 (52). Similarly, heat-killed *L. reuteri* (h-*L.reuteri*) also induced the generation and peripheral migration of Treg cells and increased the numbers of α4β7^+^ Treg cells in the spleen and joint drainage lymph nodes (**Figure 2A**). Notably, despite the immunomodulatory effects reported on mice pretreated with h-*L.reuteri*, no increase in the number of α4β7^+^ Tregs in the spleen or symptom improvement was observed in mice with collagen-induced-arthritis (CIA) (53). This might have implications for the timing of clinical application of probiotics in the future. One study hypothesized that miRNAs in breast milk may be involved in immune regulation, based on which to explore whether *L. reuteri* has a role in miRNA expression. The results showed that miRNA did affect the number of resting Treg cells and activated Treg cells in infants, but *L. reuteri* did not affect miRNAs expression (54). Studies have shown that prenatal supplementation with *L. reuteri* induced hypomethylation of CD4^+^T-cells DNA. Compared to the placebo group, the probiotic group differentially methylated probes (DMPs) were further mapped to genes. Genes associated with chemotaxis, PI3K-Akt, MAPK and TGF-β signalling are activated, which are associated with immune activation and maturation of the baby at birth, and the occurrence of atopic disease (55). Administration of *L.reuteri* ATCC 55730 didn’t change the CD3^+^ CD8^+^ T lymphocytes in the human ileum, but significantly increased the CD4^+^ T lymphocytes (56). Studies have shown that the activation of T cells promoted the growth of intestinal epithelial cells in the lamina propria of human small intestine (57). The mechanism of *L. reuteri* maintaining host intestinal health may be related to this. A previous animal study was consistent with this. Poultry colonized with Salmonella enterica serovar Typhimurium showed a significant decrease in colonization after administration of *L. reuteri* (poultry strain), and an increase in CD4/CD8 ratio was observed in the ileum of poultry. However, in the rat model of enterocolitis, *L. reuteri* increased both CD4^+^ cells and CD8^+^ cells. This is related to the decrease of intestinal mucosal barrier permeability and the enhancement of barrier function. Notably, *L. reuteri* has a structure similar to that of the mouse myelin oligodendrocyte glycoprotein (MOG) and was shown to cooperate with erysipelas bacteria to activate small intestine CD4^+^ T-cells, which have the ability to attack MOG, leading to worsening of experimental autoimmune encephalomyelitis (EAE) (58) (**Figure 2A**).

#### 2.3.2 Dendritic cells

Dendritic cells (DCs) are essential for the balance between autoimmunity and immune tolerance. *In vitro* experiments have shown that *L. reuturi* ATTC PTA 6475 and its secreted factors induced the expression of DCs maturation markers CD83 and CD86 on the surface of mouse bone marrow-derived immature dendritic cells (iDCs) and promoted the production of anti-inflammatory cytokine IL-10 by DCs. Mouse colonic organoids and animal models of acute colitis showed similar results (59) (**Figure 2B**). Similarly, *L. reuteri* induced the increased expression of IL-10 by DCs in BWF1 mice *in vitro* experiments (60). In general, *L. reuteri* and its metabolites have been shown to promote the maturation of DCs and enable their differentiation into anti-inflammatory phenotypes, which is of great significance for maintaining intestinal immune homeostasis. *L. reuteri* ATCC PTA 4659 also had a protective effect on colitis mice, but it significantly inhibited the growth of CD11b^+^CD11c^+^ DCs. It secreted the main inflammatory cytokines of colitis and made the distribution of immune cells tend to that of the control group (61). CD103 (alpha integrin) expressing CD11c^+^ DCs are essential for maintaining intestinal immune tolerance in mice (62). The lack of these DCs led to a decline in the number of Tregs and is prone to colitis (56). For mice not given *L. reuteri* 17938, about 20-30% of intestinal DCs expressed CD103, whether or not during NEC. *L. reuteri* 17938 significantly increased gut CD103^+^ DCs in WT mice, but this change was not observed in TLR2^-/-^ mice. Thus, *L. reuteri* DSM 17938 stimulated expansion of CD103^+^ DCs dependent on TLR2, thereby promoting immune tolerance (49). Oral administration of *L. reuteri* CO21 (*L. reuteri* LFCA) in newborn piglets infected with enterotoxigenic *Escherichia coli* (ETEC) not only inhibited the expression of CD40 and MHCII on the surface of DCs, but also the activation and proliferation of CD4^+^ T-cells induced by monocyte-derived dendritic cells (moDCs), thus achieving an anti-inflammatory effect (46) (**Figure 2B**). Moreover, h-*L.reuteri* was also shown to exert a regulatory effect on DCs by inducing an increase in the numbers of CD103^+^ DCs in mesenteric lymph nodes (MLNs) in mice, resulting in a reduction of the level of inflammation and improvement of disease severity (59) (**Figure 2B**). Type I interferon (IFN-I) has been known as the important link of SLE (63). Plasmacytoid dendritic cells (pDCs) produced a large amount of IFN-I (64, 65). pDC has become a key therapeutic target for SLE. Together with autoantibodies, pDCs promoted the production of immunocomplexes. These immunocomplexes were deposited in the kidneys and blood vessels, causing lupus nephritis, vasculitis, and atherosclerosis (66). Increased lupus symptoms and expression of IFN-I genes in spleen and ileum were observed after colonization of *L. reuteri* in 6-week-old GF B6 mice. This may be the result of increased pDCs in the spleen and mesenteric lymph nodes (67).

#### 2.3.3 Group 3 innate lymphoid cell

ILC3s are mainly found in the intestinal mucosa. Although they can inhibit inflammation, protect intestinal barrier function, and maintain intestinal homeostasis (68, 69), they might also drive proinflammatory responses and cause immunopathological damage under conditions of local biological disorders (70). Of note, administration of *L. reuteri* ATCC PTA 6475 was reported to reduce the number of ILC3s (**Figure 2C**), thus reducing tissue damage; however, the specific molecular mechanism remains unexplored (71). Another study found that *L. reuteri* CF48-3A promoted the production of ILC3s in neonatal mice, and induced the differentiation of IgA^+^ B–cells, eventually leading to the generation of IgA (72). However, whether the IgA of neonatal mice play a protective role against infection and enhances mucosal immunity or induces autoimmune effects remains unclear (**Figure 2C**).

#### 2.3.4 Macrophages and neutrophils

Administration of *L. reuteri* led to an increased production of nitric oxide (NO) by activated and increased numbers of macrophages, enhancing their phagocytotic ability for pathogenic bacteria. Meanwhile, *L. reuteri* inhibited the inflammation induced by bacterial infection by regulating the levels of NO (73) (**Figure 2D**). However, *L. reuteri* reduced the GBS-induced proliferation of NEs (74) (**Figure 2D**).

#### 2.3.5 Toll-like receptor 2

Toll-like receptor 2 (TLR2) recognizes the cell wall of gram-positive bacteria (75). *L. reuteri* 17938 is a gram-positive bacterium that following recognition by TLR2 mediates anti-inflammatory immune response in the following ways: First, it was demonstrated to lead to an increase in the number of Foxp3^+^ Treg cells and a decrease in that of CD4^+^CD44^+^ effector T-cells (Teffs) (49) (**Figure 2E**). The expression of CD44 on CD4^+^ T-cells is known to promote the occurrence of type 1 helper (Th1) responses and the generation of inflammatory cytokines (76). Accordingly, the activation and chemotaxis of lymphocytes to inflammatory sites was also found to be closely related to its upregulated expression (77, 78). Second, it induced the generation and activation of CD103^+^ DCs, which were characterized by the expression of co-stimulative markers CD80 and CD86 (49) that regulate the activity of T-cells (79). Of note, this DC-induced production of Foxp3^+^ Treg cells is crucial for maintaining intestinal immune tolerance (62) (**Figure 2E**). Finally, activation of TLR2 was shown to mediate the decrease in the levels of proinflammatory cytokines IL-1β and interferon-γ (IFN-γ) in mice (49) (**Figure 2E**). Notably, although *L. reuteri* 17938 has been confirmed to regulate immunity through TLR2-mediated pathways in animal models (49), oral administration of *L. reuteri* had no effects on the *in vivo* levels of TLR2 in infants with colic (52). Hence, there is still a long way to go before the mechanism of the interaction between *L. reuturi* and TLR2 can be transformed from the laboratory to the clinic.

### 2.4 Regulation of cytokines for improving intestinal mucosal barrier structure and permeability

Several studies have demonstrated that *L. reuteri* inhibited the production of proinflammatory cytokines, such as tumor necrosis factor-α (TNF-α), IL-1β, IL-6, and IL-8 (42, 43, 48, 59, 71), and that of CXCL10, CXCL1, and CCL2 chemokines, which recruit inflammatory cells (80), while it promoted the production of the anti-inflammatory cytokine IL-10 (53, 59), thus stabilizing the expression of TJs and E-cadherin and reinforcing the intestinal barrier function. Likewise, *L. reuteri* LFCA inhibited the expression of nuclear factor kappa-B (NF-κB)-mediated p65 and phosphorylation of inhibitor of NF-κB(IκB)α(IκBα) and nuclear transfer of p65, thereby inhibiting the transcriptional activation of inflammatory genes, as well as suppressing the production of proinflammatory factors, such as TNF-α, IL-6, and IL-1β (46) (**Figure 2G**). A significant decrease in the expression of LPS-induced TNF-α and IL-6 was observed in IPEC-J2 porcine IECs treated with *L. reuteri* I5007. However, this inhibition was dependent on TLR4 and related to LPS and time (38) (**Figure 2G**). Recent studies have shown that *L. reuteri* regulates the levels of TNF-α in a strain-dependent manner. For instance, *L. reuteri* ATCC PTA 6475 inhibited the activation of human monocyte MAPK, which in turn inhibited the activation of transcription factor c-Jun and AP-1, ultimately restraining the production of TNF-α (81) (**Figure 2G**). Similarly, *L. reuteri* BM36301 showed a gender preference in regulating the levels of TNF-α, as it significantly reduced the expression of TNF-α in female mice compared with that in male mice (82) (**Figure 2G**). Moreover, *L.reuteri* 5289 inhibited the generation of DCs and associated production of IL-12, whereas induced the expression of IL-10, which is associated with prolonged phosphorylation of extracellular regulated protein kinases1/2 (ERK1/2) in the MAPK pathway (83) (**Figure 2G**). A decrease in the levels of TGF-β in breast milk was observed after mothers in late pregnancy were provided a diet supplemented with *L.reuteri*. Notably, this might have been related to the decrease in the prevalence of allergy and IgE-related eczema in newborns in the first 2 years of their life (84) (**Figure 2G**). An engineered *L. reuteri* strain induced the production of IL-22 in the intestinal tract, hence mediating the expression of lectin regenerating islet-derived 3 gamma (REG3G), which, in turn, inhibited bacterial translocation, playing a protective role in liver (85) (**Figure 2G**). Notably, gene editing of *L. reuteri* strains produced better immune protection, providing novel ideas for the application of *L. reuteri*. Finally, *L. reuteri* has also been reported to regulate the expression of chemokines. For instance, CCL2 is known to be involved in pathological damages induced by stressor-enhanced infective colitis. Administration of *L. reutri* inhibited the expression of CCL2, thus reducing the infiltration of monocytes/macrophages in colon and secretion of inflammation-related factors (86).

### 2.5 Promotion of synthesis and secretion of immunoglobulin

Administration of *L reuteri* CF48-3A—derived from human milk—to pregnant C57BL/6(B6) mice induced the synthesis of IgA in mice offspring during their early life through vertical transmission from the vagina and milk. The mechanism by which this intestinal symbiotic microorganisms of maternal origin increased the synthesis of IgA in pups remains unclear; however, researchers found that both T-cells and RORγt^+^ ILC3s were dispensable in newborn mice (72). Of note, this maternal microbe-promoted synthesized IgA did not provide protection against intestinal infection by common pathogens such as *Salmonella typhimurium (S. typhimurium)* or *enterohemorrhagic E.coli* (72), and its function in pups remains unknown. Another study found that administration of *L. reuteri* FN041 increased the synthesis of secreted IgA (sIgA) in the ileum of mice (**Figure 2F**), especially in female mice (87). Vaccinating newborn mice with *L. reuteri* CRL1324 prior to GBS infection not only stimulated the production of IgA, but also that of IgG, which is related to anti-infection functions (74) (**Figure 2F**). Changes in microbial structure caused by *L. reuteri* have also been associated with the production of Abs. For example, an increase in the numbers of lactobacilli and enterococci caused by administration of mixed strains of *L. reuteri* were correlated with increased levels of IgE (88) (**Figure 2F**). The function of the Abs that promoted by *L. reuteri* is uncertain. Further studies are needed to determine whether it functions as a protective or an autoimmune factor. Sialylation of IgE is a key pathogenic link in allergies (89). Removal of sialic acid from IgE or de-sialic acid glycoprotein reduced allergic reactions (90). Whether *L. reuteri* can regulate glycosylation of IgE to improve allergy is the next research direction.

## 3 Applications of *L. reuteri* in the prevention and treatment of immune-related diseases

### 3.1 Atopic dermatitis

Atopic dermatitis (AD) is associated with an impaired skin barrier, skin microbial dysbiosis, and immune dysfunction (91–96). Skin microorganisms have an important role in the pathogenesis of AD, with increased colonization by *Staphylococcus aureus (S.aureus)* triggering inflammation or worsening AD (91, 97–100). The pathogenesis of AD has not only been related to skin microorganisms, but also to gut microbiota. Notably, the gut microbiome of infants with AD and their mothers differed markedly from that of healthy mothers and infants in the abundance of specific taxa (101). Accordingly, administration of oral probiotics in infants and children over 1 year old of age exhibited clear efficacy in reducing the incidence of AD and improving AD symptoms (102). Similarly, oral administration of probiotics in young patients with moderate AD significantly decreased the Scoring Atopic Dermatitis Index (SCORAD) values, an index assessing the severity of AD, and led to a reduction in the use of topical glucocorticoids (GCs) (103). Animal studies have shown that administration of probiotics to AD mice resulted in a decrease in the levels of the intestinal inflammatory marker fecal calprotectin (FC), reduced the levels of inflammatory factors, and ameliorated the incidence of skin lesions (104). These findings were also confirmed in clinical studies. After administration of a probiotic mixture containing *Lactobacillus* and *Bifidobacterium* for 4 weeks in probiotics group, both the SCORAD index and FC levels were decreased, accompanied by a decrease in the number of microbial species (105). Although a causal relationship between alterations in the composition of the gut microbiome and AD symptoms is not clear, the modulation of gut microbiota by oral or topical skin probiotics was shown to ameliorate AD, thus providing new ideas for probiotic intervention strategies against AD in the future.

The role of *L. reuteri* in the treatment of AD has only been recently noted. In an animal experiment, *L. reuteri* Fn041 derived from breast milk was found to ameliorate symptoms, such as skin swelling and inflammatory cell infiltration, in AD mice by modulating the ratio of Th1 and Th2 secreted cytokines, promoting the generation of Treg cells, and modulating the intestinal flora to increase the abundance of *Lactobacillus* and *Akkermansia* (50) (**Table 1**). Another study found that its anti-inflammatory effect was achieved through the activation of retinol metabolism and PPAR signaling pathways in Peyer’s patches. Notably, they argued that administration of late gestational dams with *L. reuteri* Fn041 and continued supplementation until weaning had better effect than that of supplementation after weaning (**Table 1**). This result emphasized the role of *L. reuteri* transmitted vertically by the mother in shaping the intestinal microbiota and strengthening the intestinal barrier of the offspring (107). AD is well-known to be an inflammation-induced disorder mainly mediated by Th2 cells (121, 122). Thymus stromal lymphopoietin (TSLP) is an important cytokine that mediates Th2 inflammatory responses (123, 124). *L. reuteri* DYNDL22M62 inhibited the production of TSLP and caused a decrease in the levels of Th2 cytokines (108), thus hindering the occurrence and progression of inflammation in AD. These studies showed that *L. reuteri* DYNDL22M62 metabolized Try into indoles, resulting in an increase in the proportion of intestinal flora that produce indole LAC and increasing the expression of AHR (108), thereby inhibiting intestinal inflammation and regulating immunity (125, 126). Furthermore, *L. reuteri* DYNDL22M62 reduced the production of IgE and improved AD symptoms (108) (**Table 1**). Oral administration of *L. reuteri* ATCC 55730 to children with AD for 8 weeks reduced the production of IL-4 and regulated the levels of cytokines in intestinal and extraintestinal tissues (106) (**Table 1**). However, the study did not clarify the correlation between the changes in the levels of related cytokines caused by *L. reuteri* ATCC 55730 and the improvement in AD symptoms. Administration of an oral probiotic mixture significantly reduced SCORAD index and steroid use in children with moderate AD (103). Besides, studies have found that continuous administration of an oral probiotic mixture in pregnant and lactating mothers and infants significantly reduced the incidence of AD in children (127). These findings suggest that the use of *L. reuteri* mixed with other strains might greater benefit patients with AD. However, currently, no study has compared the efficacy of mixed probiotics containing *L. reuteri* to that of single probiotics in the prevention and treatment of AD. The current view is that *L. reuteri* can improve AD, but only few randomized controlled trials (RCTs) have been performed in animals and the clinic, to date. In the future, large sample RCTs should be conducted on the basis of selecting appropriate strains. As the onset of AD is also correlated to imbalances in skin microecology (91, 128), regulating skin microecology might also be a way to prevent and treat AD. However, to date, no study has explored the application of *L. reuteri* as a human skin probiotic. In the future, the use of emollient cream or slow-release agent containing single or mixed probiotics might be a potential way to optimize the application of probiotics for the prevention and treatment of AD.

**Table 1:** Applications of *L. reuteri* in diseases.

Disease	Strains	Participants	Intervention	Mechanism of action	Outcomes	Ref
AD	ATCC 55730	Children aged 4–10 years (n = 26)	10^8^ CFU/d, 8 weeks	Increased levels of IFN-γ and reduced levels of IL-4 in exhaled breath condensate	No changes in SCORD Index mean values	(106)
	Fn041	7-week-old BALB/c mice during late gestation and lactation (n = 6), 3-week-old infant BALB/c mice (n = 8)	10^9^ CFU/d from 1 week before parturition to weaning	Reduced numbers of mast cells and eosinophils; increased numbers of Tregs cells; increased levels of IL-12; reduced levels of IL-4; reduced levels of IgE; enrichment of *Lactobacillus* and *Akkermansia* species; reduced numbers of *Alloprevotella* spp; increased numbers of *Limosilactobacillus reuteri* in breast milk	Reduced redness, swelling, and relative thickness of ear; decreased incidence of AD	(50)
		7-week-old BALB/c mice during late gestation and lactation, infant (n = 6), 3-week-old infant BALB/c mice (n = 12)	Maternal mice with 10^9^ CFU/d from day 3 before parturition to weaning, infant mice with 10^9^ CFU/d for 10 days	Reduced levels of plasma OVA-specific IgG1/IgG2a; reduced levels of IL-4, IL-33, and TSLP; increased numbers of splenic Tregs; decreased numbers of eosinophils and mast cells; increased numbers of *Limosilactobacillus*, *Faecalibacterium*, and *Akkermansia*; activation of retinol metabolism and PPAR signaling pathway; downregulation of pathways associated with asthma, autoimmune thyroid disease, and SLE.	Reduced swelling and relative thickness of ear; increased height of ileal villi and ratio of ileal villus height to crypt depth; decreased incidence of AD	(107)
	DYNDL22M62	6-week-old C57bl/6 mice (n = 6)	10^9^ CFU/d, 3 weeks	Reduced levels of IgE; reduced expression of TSLP, IL-4, and IL-5; reduced Th2 type responses; increase generation of ILA and IPA; increased numbers of *Romboutsia* and *Ruminococcaceae* NK4A214; reduced numbers of *Dubosiella*	Elevated tryptophan metabolism; reduced ear swelling and skin lesions; alleviated AD	(108)
Asthma	Five-strain mixture including CCFM1072 (FSDLZ13M6),DYNDL2-16, CCFM1040 (YN-DL-1-3), GDLZ10-5, and FZJTZ20M3	4–5-week-old female BALB/c mice (n = 6–7)	(10^9^ CFU)/strain/d, 1 week before the first sensitization until the end of the experiment, 6 weeks	Reduced levels of total IgE and HDM-IgG1; reduced levels of IL-5 and IL-13; enrichment of *Lactobacillus* and *Enterococcus* species; regulation of gut microbial function toward butyrate generation	Decreased airway inflammation score; decreased incidence of asthma	(88)
	ATCC 23272	8–9-week-old male BALB/c and Toll-like receptor 9–deficient mice	10^9^ CFU/d, 9 days	Reduced numbers of eosinophils; reduce levels of TNF, MCP-1, IL-5, and IL-13; dependence on TLR9 and increased activity of indoleamine 2,3-dioxygenase	Reduced airway hyperresponsiveness; attenuated asthmatic response	(109)
	DSM 17938	Children and adolescents aged 6–17 years with mild to moderate asthma (n = 14)	10^8^ CFU/d, 60 days	/	Increased ACT scores; reduced number of symptoms and wheezing	(110)
		Adults with mild allergic asthma, 8 women and 7 men , mean age of 27 years (n = 15)	10^9^ CFU/d, 4 weeks, followed by a second treatment period with 10^9^ CFU/d for 4 weeks and then by 4–5 weeks of washout period	/	No differences in airway nerves, smooth muscle, sputum inflammatory cells, skin responses, or T-cell responses	(111)
	CCFM1040	Adults aged 18–60 years with at least a 1-year-long history of rhinitis or asthma or both (n = 4)	10^9^ CFU/d , 8 weeks	Decreased numbers of Proteobacteria phylum, Escherichia_Shigella genus, and Intestinibacter order; promotion of mineral absorption and apoptosis; inhibition of novobiocin biosynthesis; altered biological pathways associated with the metabolism of carbohydrates, energy, lipids, cofactors, and vitamins, xenobiotic biodegradation and metabolism, and immune system	Decreased TSS, RQLQ, 3 nasal scores in TSS (nasal congestion,watery eyes, and rhinorrhea) and mean sneezing score; improved sleep and non-nose/eye symptoms; increased ACT score; enhanced control in patients with asthma	(112)
NEC	DSM 17938	5-day-old newborn C57BL/6J (WT) mice and B6.129-TLR2^tm1kir^/J (TLR2^-/-^) mice	NEC-induced+LR 17938-fed (WT: n = 21; TLR2^-/-^: n = 12), 10^6^ CFU/g body/d/pup, 4 d	Reduced numbers of activated effector CD4^+^ T-cells; increased numbers of Foxp3^+^ Tregs; activated tolerogenic DCs by TLR2; decreased levels of IL-1β and IFN-γ	Decreased incidence and severity of NEC	(49)
		Preterm infants of ≤32 weeks gestational age and birth weight ≤1500 g (n = 400)	10^8^ CFU/d, from first feeding to discharge	/	No effect in incidence and mortality of NEC; reduced frequency of proven sepsis, rates of feeding intolerance, and duration of hospital stay	(113)
		Infant Sprague-Dawley rats	NEC+17938 (n = 38), NEC+4659 (n = 36), formula+17938 (n = 22), formula+4659 (n = 17), 10^6^ CFU/g body/d, 3 days	Downregulated levels of TLR1, TLR4, IL-1β, IL-6, TNF-α, and nfrκb; upregulated levels of IL-10 and Nfκbib; inhibited MAPK8IP3; inhibited LPS-induced IκB phosphorylation	Increased survival rate; decreased incidence and severity of NEC	(114)
	ATCC PTA 4659			Downregulated levels of TLR1, TLR4, IL-1β, IL-6, TNF-α, and nfrκb; upregulated levels of IL-10 and Nfκbib; inhibited production of myelin and lymphocyte protein; inhibited LPS-induced IκB phosphorylation		
SLE	Unknown	TLR7.1 Tg C57BL/6 mice and WT B6 mice	Gavage daily	Translocation depending on TLR7; increased abundance and translocation of *Lactobacilli*; increased numbers of pDCs and leukocyte recruitment; increased IFN signaling	Increased splenomegaly and hepatomegaly; worsened IMQ-induced anemia and gut permeability; increased lupus-related pathogenesis and systemic autoimmunity	(115)
	GMNL-263	16-week-old BWF1 mice (n = 5)	10^8^ cells/mL/d, up to week 28	Reduced number of TUNEL-positive cells; decreased levels of TNF-R1, FADD, MMP-9; increased levels of p-AKT	Decreased cardiac apoptosis and fibrosis	(116)
	GMNL-89	6-week-old BWF1 mice	10^9^ CFU/d, 12 weeks	Reduced levels of hepatic MMP-9, CRP, and iNOS; reduced levels of IL-1β, IL-6, and TNF-α; suppressed MAPK and NF-κB; reduced hepatic lymphocyte infiltration; reduced numbers of TUNEL-positive cells and levels of cleaved caspase-3; Reduced ratios of p-ERK:ERK, p-P38:P38, and p-JNK:JNK; reduced expression of IKK and NF-κB	Decreased hepatic apoptosis and inflammation	(117)
	DSM 17509	4-week-old female BWF1 mice (n = 6–11)	10^8^ CFU/biw, 10 months	Increased levels of IL-10 and IL-12	Delayed lupus onset; increased survival	(60)
RA	CCFM 8631 and CCFM 14	6-week-old female Wistar rats	2.5 × 10^8^ CFU/d, 2 weeks before CIA induction and continued for another 7 week	Reduced levels of serum anti-CII IgG and anti-CII IgG2b; attenuated increase in the levels of IL-6 and TNF-α; decreased levels of IL-1β, IFN-γ, and IL-12; downregulated levels of IL-10; enriched *Verrucomicrobia* and *Bifidobacterium* species; restored decrease in numbers of *Clostridium* species. increased levels of SCFAs; enriched microbial metabolic functions	Decreased ankle swelling; attenuated arthritis	(118)
	MM2-3 (ATCC PTA 4659)	6–8-week-old female DBA/1J mice (n ≥ 6)	Gavage at days 7–21 or days 21–35, 14 days	Reduced levels of serum CII-specific IgG, IL-6, and CXCL1; increased levels of IL-10; increased levels of Tregs, CD4^+^IL-10^+^ cells, CD103^+^ dendritic cells, and α4β7^+^ Tregs; promoted peripheral migration of Tregs	Decreased incidence, severity, and progression of arthritis	(53)
MS	Unknown	GFAP-AHR deficient mice	/	Conversion of dietary Trp into AHR agonists via TnAse dependent and independent pathways	Regulated astrocyte function	(119)
	Unknown	10-week-old female WT C57BL/6 mice	10^8^ CFU/d, 20 days	Reduced numbers of CD3^+^ T-cells, CD68^+^ macrophages, Th1, and Th17 cells; reduced levels of IL-17 and IFN-γ; restored diversity of gut microbiota; decreased splenocyte proliferation; reduced numbers of *Proteobacteria* and *Deferribacteres*; reduced numbers of *Anaeroplasma* and *Rikenellaceae*; increased numbers of *Bacteroidetes*; increased numbers of *Prevotella* and S24-7	Decreased severity of EAE. regulated microbial dysbiosis	(120)
	H4 and LMG 18238	5–7-week-old female GF C57BL/6 mice	Colonization	Cooperation with OTU0002 (*Erysipelotrichaceae*); upregulated expression of UvrA; cross-reaction with and activation of Ki67^+^ proliferating CD4^+^ T-cells; activation of antigen-specific Th17 cells	Aggravated inflammation in spinal cord	(58)

"/" indicates that the study does not have information on the mechanism of action or Intervention. OVA, ovalbumin; TSLP, thymic stromal lymphopoietin; PPAR, Peroxisome Proliferator Activated Receptor; ZO-1, zonula occludens-1; ILA, indole lactic acid; IPA, indole propionic acid; MCP-1, Monocyte Chemoattractant Protein-1; TLR9, Toll-like receptor 9; DCs, dendritic cells; Tem, Effector memory T; nfrκb, nuclear factor related to κB-binding protein; Nfκbib, NF- κB inhibitor-β; MAPK8IP3, mitogen-activated protein kinase 8 interacting protein 3; IκB, inhibitor of NF-κB; pDCs, Plasmacytoid Dendritic Cells; TNF-R1, tumor necrosis factor receptor 1; FADD, Fas-associated protein with death domain; MMP-9, matrix metalloprotein-9; p-AKT, phospho-AKT; CRP, C reactive protein; iNOS, inducible nitric oxide synthase; MAPK, mitogen-activated protein kinase; p-ERK, phosphorylated extracellular signal-regulated kinase; p-JNK, phosphorylated c-Jun N-terminal kinase; p-P38, phosphorylated P38; IKK, IκB kinase; SCFAs, short chain fatty acids; Trp, Tryptophan; GFAP, glial fibrillary acidic protein; AHR, Aryl Hydrocarbon Receptor; TnAse , tryptophanase; GM-CSF, granulocyte macrophagecolony stimulating factor; UvrA , UvrABC system protein A; ArAT, aromatic amino acid aminotransferases; fldH, D-lactate dehydrogenase; AmiE, aliphatic amidase E; SCORAD, Severity scoring of atopic dermatitis; ACT, the Asthma Control and Test; FEV, Forced Expiratory Volume; TSS, total symptom score; RQLQ, Rhinoconjunctivitis Quality of Life Questionnaire; IMQ, Imiquimod ; EAE, Experimental autoimmune encephakmyelitis ; CNS, Central Nervous System.

### 3.2 Asthma

Asthma is a chronic airway inflammatory disease (129). Both Th2 cytokine-mediated inflammation and increased levels of IgE are important links in the pathogenesis of asthma (130, 131). Notably, seasonal exacerbation of asthma has been reported to be synchronous in time with changes in the composition of respiratory tract microbiota (132). Further, *Streptococcus*, *Haemophilus*, and *Moraxella* are believed to cause the deterioration of airway inflammation (133–135). Inhaled GCs are commonly used for the treatment of asthma. The application of these GCs leads to changes in the composition of airway microbiota, which have been correlated with the number of eosinophils and neutrophils in the sputum (136). In recent years, the discovery of the gut-lung axis also provides a possibility to explain the relationship between intestinal flora and lung health and disease. Accordingly, a possible mechanism is an intestinal ecological imbalance. Microorganisms and their metabolites might be transferred from the damaged intestinal barrier to the lung through blood or lymph circulation, causing lung inflammation and asthma-related symptoms (137–140). Additionally, intestinal microflora is crucial in the immune development of infants (141). Of note, retardation in intestinal microbial growth caused immune maturation disorder, affecting the immune responses of the lung, causing intolerance or overreaction to allergens (142). The lack of microbiota in the first year of life of infants born to asthmatic mothers confirmed this finding, as indicated by the increased risk of asthma in these children at the age of 5 (142).

As a dietary supplement, probiotics reshape the composition of intestinal microorganisms and might thus become potential candidates for early intervention in patients with asthma. Probiotics have been reported to not only have a positive impact on the health of newborns, but also on tackling allergic diseases of children over 14 years old (143). Although the conditions under which probiotics play their ideal role remain unclear, their administration has been shown to result in improvement of asthma-related parameters and symptoms in patients participating in clinical trials. For example, probiotics improved the anti-inflammation lipid depletion in the placebo group (141), significantly reduced the alveolar NO concentration of patients (P = 0.038), greatly relieved their symptoms (P = 0.001), and improved their score in the asthma control test (ACT) (P = 0.023). In addition, they increased the diversity of microorganisms, gene expression in potentially beneficial bacteria, such as *Bifidobacteria*, and the production of microbial active metabolites (144). Animal studies showed that probiotics reduced the levels of Th2-secreted cytokines IL-4, IL-5, IL-13, whereas increased those of the Th1-secreted cytokine, IFN-γ. They also inhibited the aggregation and inflow of eosinophils in the lung, and reduced the serum levels of IgE and airway hyper-responsiveness (145). Apart from this, they inhibited the deposition of lung collagen and proliferation of goblet cells, and reduced airway remodeling (146). The role of probiotics in regulating the levels of serum cytokines, lowering of the levels of IgE, and reducing eosinophil infiltration in patients with asthma was also confirmed in another clinical study (147). In this study, although probiotics had a positive effect on asthma-related immune biomarkers, the clinical symptoms of asthma were not improved (147). The inconsistent effect of probiotics on asthma-related parameters and clinical symptoms could be relevant to the dosage of probiotics. Hence, designing experiments where different dosages will be tested are necessary to clarify whether a correlation exists between dosage and effect. Thus, at present, probiotics should be carefully used in the prevention and treatment of asthma, especially in infants, considering all safety precautions. A large number of samples and the selection of appropriate strains and combinations are needed to further clarify whether probiotics are efficacious in the prevention and treatment of asthma and whether they can replace asthma drugs.

Owing to their great variety, some probiotics might become harmful under certain conditions. Thus, selecting the appropriate probiotic strains for targeting a specific disease is crucial. Some studies have compared the effects of *L. reuteri*, *L. rhamnosus*, and other strains on mice with asthma induced by the house dust mite (HDM), and observed that *L. reuteri* exhibited the best effect. This confirmed the core position of *L. reuteri* in regulating intestinal flora to affect lung immunity. Briefly, *L. reuteri* improved the airway inflammation index and decreased the total serum levels of IgE, HDM-IgG1, and Th2 cytokines, suppressing airway inflammation. Apart from this, it increased the abundance of *Lactobacillus* and *Enterococci* in the intestine and affected the production of total IgE and IL-13. Moreover, it increased the production of butyric acid, a microbial metabolite, by reshaping the intestinal microbial structure (88) (**Table 1**). The findings of this study were consistent with those in which oral *L. reuteri* inhibited eosinophil infiltration in the respiratory tract of mice, reducing the levels of cytokines, such as TNF, monocyte chemoattractant protein-1 (MCP-1), IL-5, and IL-13 in the lung, as well as reducing airway hyper-responsiveness (109) (**Table 1**). A clinical study of 30 patients aged 6–17 years—where patients were divided into 2 groups, those that were administered beclomethasone only, and those that were administered beclomethasone and *L. reuteri DSM* 17938 10^8^ colony forming units/day (CFU/d)—revealed that the asthma-related symptoms in the probiotics group were improved and the score of ACT and peak expiratory flow (PEF) were increased after 2 months (110) (**Table 1**). Similarly, *L. reuteri* CCFM1040 also improved ACT and led to an improved control of asthma symptoms (112) (**Table 1**). In contrast, a clinical study found that the use of *L. reuteri* DSM 17938 10^9^ CFU/d in adults with an average age of 27 years did not improve respiratory inflammation and symptoms, skin allergy, or T-cell responses (111) (**Table 1**). The discrepant effects of *L. reuteri* DSM 17938 in these two clinical trials might have been affected by age and dosage. Of note, the form of *L. reuteri* also affected its effect on asthma. For example, live *L. reuteri* exhibited an anti-inflammatory role and reduced airway hyper-responsiveness, whereas inactivated *L. reuteri* did not have this effect (109). In general, the administration of *L. reuteri* has shown beneficial effects on relieving asthma symptoms, and thus might become a potential candidate for the adjuvant treatment of asthma. Identification of the ideal strain, dosage, duration, and specific mechanism of action of *L. reuteri* in the prevention and treatment of asthma is warranted in the future.

### 3.3 Necrotizing enterocolitis

Necrotizing enterocolitis (NEC) is a fatal acute inflammatory disease of the intestine that tends to occur in newborns, especially preterm infants (148), with a high incidence rate (149, 150). The risk factors of NEC include premature delivery, no breastfeeding (151), increased antibiotic exposure (152), and intestinal microbial ecological imbalance (153). Notably, 16S rRNA gene sequencing analysis found that the abundance of *Proteus* was increased in infants with NEC, whereas that of *Bacteroides* and *Firmicutes* was decreased (154). Some studies also found that the increase in the numbers of *Clostridium* (155, 156) or *Klebsiella* (157, 158) occurred before the diagnosis of NEC in premature infants. Changes in the abundance of *Lactobacillus*, *Staphylococcus* (159), and *Streptococcus* (160) have also been implicated in the occurrence and development of NEC. Although, these changes in the composition of intestinal microorganisms among children with NEC exhibit individual differences (159), their common feature is the low diversity of microorganisms (156, 159). Therefore, identifying specific microorganisms as biomarkers for the early diagnosis of NEC and applying prompt therapeutic approaches to reduce the incidence and severity of NEC, is imperative. Compared with the control group, sterile mice receiving fecal flora transplantation from patients with NEC showed more severe intestinal pathological damage (154). Of note, in another study, premature and cesarean piglets that received fecal filtrate transplantation from healthy piglets did not develop NEC (161). These evidence revealed that using intestinal microbiota as a therapeutic modality is of great significance in the prevention and treatment of NEC, and highlighted the potential use of probiotics as the next generation of promising therapies for NEC.

There is evidence to support that administration of single strains or combinations of multiple strains of *Lactobacillus* or *Bifidobacterium* has more advantages than that of other strains or combinations in reducing the incidence rate of NEC in preterm infants, by improving severity and reducing mortality (162, 163). However, it should be noted that the pathogenic or protective effect of probiotics on NEC is strain-dependent. Different strains of the same species might have distinct effects on NEC. For example, *Enterococcus faecalis (E. faecalis)* 224 improved the intestinal pathological damage of NEC rats, whereas BB70 and BB24 worsened NEC (164). Of note, the use of probiotics not only inhibited the growth of pathogenic and opportunistic pathogens (165), but also reduced the feeding intolerance of premature infants, which is of great significance to the nutrition and subsequent growth and development of the nervous system (163). In general, oral probiotics prevented the occurrence of NEC and improved the condition especially in terms of prevention in animal models and clinical trials, albeit not without certain risks. Although probiotics promote the health of the body, premature infants might be sensitive to them as their immune system is not yet mature. Further research is needed to evaluate the short-term and long-term effects and potential safety risks of the administration of probiotics to premature infants.

As a member of *Lactobacillus*, *L. reuteri* was studied for its prophylactic and therapeutic effect in NEC. Notably, TLR4, TLR2, and NF-κB are highly expressed in the intestine of experimental NEC animal models, mediating intestinal injury, as confirmed in many experiments (151, 166–168). Both *L. reuteri* DSM 17938 and *ATCC PTA 4659* downregulated the expression of TLR4 and NF-κB and that of the pro-inflammatory cytokines IL-6, TNF-α, and IL-1 β, whereas upregulated the expression of the anti-inflammatory cytokine IL-10, inhibiting the occurrence and severity of NEC in neonatal rats. Among them, *L. reuteri* DSM 17938 was found to be more effective (114) (**Table 1**). Mechanistically, the improvement of experimental NEC by *L. reuteri* DSM 17938 involved the regulation of immune cells. In particular, *L. reuteri* DSM 17938 reduced the numbers of CD4^+^ effector T-cells, whereas increased those of Foxp3^+^ Tregs (49, 51) and tolerogenic dendritic cells (tolDC) in intestinal mucosa. These phenomena were observed in wild-type (WT) mice, but not in TLR2^-/-^ mice, suggesting that the anti-inflammatory protective effect of *L. reuteri* DSM 17938 might be related to the TLR2 signaling pathway (49) (**Table 1**). Moreover, *L. reuteri* DSM 17938 reduced intestinal inflammation by inhibiting the oxidative stress reaction and enhancing the intestinal antioxidant capacity of NEC mice (47) (**Table 1**). However, to date, there have only been a few clinical studies in humans. A retrospective cohort study analyzed the medical data of 311 extremely low-birth weight infants (ELBWI) with birth weight ≤ 1000 g, 232 of whom did not use probiotics, and 79 of whom that received 0.1 mL (10^8^ CFU/0.18 mL) of probiotics containing *L. reuteri* every day after birth. The incidence of NEC was decreased from 15.1% (35/232) to 2.5% (2/79) (169) (**Table 1**). In another RCT, *L. reuteri* DSM 17938 was administered to 400 very low-birth weight infants (VLBWI) (≤ 1500 g) at 10^8^ CFU/d. Although no differences were detected in the incidence and mortality of NEC between the intervention and placebo groups, probiotics significantly reduced the incidence of delayed sepsis and feeding intolerance, and shortened the length of hospital stay (**Table 1**). However, this trial did not further randomize premature infants with weight ≤ 1000 g (113). The lower weight group was more likely to present positive results, which might be the reason for the differences observed in the experimental results. Current evidence has supported that the application of *L. reuteri* in animal models reduced the incidence rate of NEC, suggesting its potential protection against NEC in clinical trials. However, large sample, multicenter RCTs need to be conducted in the future to verify the preventive effect of *L. reuteri* on NEC pathogenesis in preterm infants. As the combination of *Lactobacillus* and *Bifidobacterium* is currently the recommended combination of probiotics for the prevention and treatment of NEC, the experimental combination of *L. reuteri* and *Bifidobacterium* can be tested in the future to verify its efficacy against NEC. Further understanding of the pathogenesis of NEC and exploration of whether *L. reuteri* can act on key targets involved in its pathogenesis is the direction of our efforts.

### 3.4 Systemic lupus erythematosus

Systemic lupus erythematosus (SLE) is a complex autoimmune disease. Recent studies have observed changes in the composition of the intestinal microbiome and its metabolic functions in SLE (170–174). Sequencing analysis of intestinal microorganisms in patients with SLE showed low diversity and unbalanced distribution of species ratio. The metagenome-wide association study (MWAS) and the genome-wide association study (GWAS) showed that the abundance of *Streptococcus intermedius* and *Streptococcus anginosus* increased in the gut of SLE patients (175). The abundance of certain species increased in untreated SLE patients, including the *Clostridium species* ATCC BAA-442 as well as *Atopobium rimae, Shuttleworthia satelles, Actinomyces massiliensis, Bacteroides fragilis, and Clostridium leptum*. After treatment, these microorganisms decreased. The proinflammatory ability of some microbial peptide structures of SLE-enriched species was also confirmed by functional validation assays (176). An animal trial in 2020 found that the transfer of the gut microbiota of triplecongenic (TC) lupus-prone mice to germfree congenic C57BL/6(B6) mice reduced autoimmune severity. Metabolomics results showed that the distribution of tryptophan metabolites in feces of TC mice changed. Further study showed that high dietary tryptophan could aggravate the pathology of TC mice and the reduction of dietary tryptophan changed the intestinal microbiota of TC mice and B6 mice, suggesting that tryptophan metabolism might lead to immune activation of SLE by changing the microbiota structure (176). Another animal study in 2022 found defective TCR signaling proliferation of segmented filamentous bacteria in the gut of B6SKG mice, driving Th17 cell differentiation and causing autoimmunity in SLE (177). The intestinal microecology of patients with SLE is out of balance, and characterized by decreased microbial diversity, which is particularly noticeable in active patients (178). Changes in the content of specific species, such as increased numbers of *Streptococcus*, *Campylobacter*, and *Velocilla*, promote the arrival of the active phase of lupus, whereas increased numbers of *Bifidobacterium* prolong the remission phase of SLE (179). Patients with SLE can be distinguished from those with rheumatoid arthritis (RA) and healthy people by analyzing the differences in the composition and metabolic functions of intestinal microorganisms. These criteria can also further distinguish the active from the remission period of SLE (179). Transplantation of the fecal flora of SLE mice or patients to sterile mice significantly increased the level of anti-dsDNA, stimulated the inflammatory immune response, and increased the expression of SLE susceptibility genes (171, 180). After receiving *Ruminococcus(RG)* transplantation from patients with lupus, C57BL/6 mice had increased levels of anti-RG antibodies and intestinal permeability, especially female mice (157).In addition, transplantation of fecal flora from SLE patients also affected the metabolism of histidine in sterile mice, resulting in increased production of histamine, which might also be involved in the pathogenesis of SLE (171). In conclusion, the administration of probiotics for the treatment of microbial ecological imbalance in patients with SLE might be a new treatment direction that can bring hope to patients with SLE.

The number of probiotics used in SLE-related experiments has been limited, with *Lactobacillus* being the most commonly used genus. Notably, SLE affects multiple systems, and is often associated with cardiovascular and renal diseases (181–183). In the case of SLE-related renal injury, the use of probiotics reduced the levels of serum anti-dsDNA in female mice of a hypertensive lupus model. In addition, the level of inflammation in renal tissues was decreased, mainly manifested by the decrease in the numbers of Th1 and Th17 cells and levels of proinflammatory cytokines. Moreover, long-term use of probiotics alleviated renal injury and improved renal function, by inhibiting the activity of NADPH oxidases (NOX), thereby reducing the level of oxidative stress (184). In the case of SLE-related cardiomyopathy, administration of probiotics inhibited cardiac fibrosis and anticardiomyocyte apoptosis, while led to the significant thickening of the ventricular wall and the restoration of the structure of mildly changed cardiomyocytes to a certain extent. These findings were further confirmed by the decrease in the number of apoptotic cardiomyocytes (185). Regarding SLE-related vascular diseases, probiotics might play a role in preventing endothelial dysfunction. For example, the sensitivity of mouse aorta to acetylcholine, which depends on the levels of NO produced by endothelial cells, was increased after administration of probiotic (186). The excess of reactive oxygen species (ROS) is a pathogenic factor for cardiovascular diseases (187). Moreover, the excessive production of ROS is also known to be combined with increased levels of NO and inactivate vasodilator NO (188). Probiotics inhibited the activity of nitrogen oxides and reduced the production of ROS, thus maintaining the balance of ROS/NO and restoring the normal function of vascular endothelium (186). Based on their protective effect on endothelial dysfunction, probiotics might also have potential effects on preventing hypertension (186). Apart from their positive protective effect on SLE-related multisystem complications, combinations of probiotics and drugs were also reported to enhance the efficacy of drugs. A study found that probiotics combined with prednisolone pretreatment or -treatment reduced the serum levels of antinuclear antibody (ANA), anti-dsDNA antibody, and anti-RNP antibody in SLE mice (189). These autoantibodies are key pathogenic factors of SLE-related organ or tissue damage (190–193). In addition, a Th17/Treg imbalance is an important marker of inflammation progression during the pathogenesis of SLE (194–196). Hence, regulation of the Th17/Treg balance is of great significance for the protection from SLE-related complications (184, 197, 198). Tacrolimus (Tac) is an immunosuppressive agent commonly used in the treatment of SLE (199–201), and also known to inhibit Treg cells (202). A study found that compared with using Tac or probiotics alone, the combination of Tac and probiotics reduced the serum levels of autoimmune antibodies in MRL/lpr mice. In addition, both *in vivo* and *in vitro* experiments revealed an increase in the numbers of Treg cells and a decrease in those of Th17 cells. Based on this, the authors of the study suggested that probiotics could be used as the synergist of Tac for the regulation of the Th17/Treg balance in the SLE mice model (198). Various animal experiments have shown that probiotics might have anti-inflammatory and potential immunomodulatory effects in the prevention and treatment of SLE-related nephritis, cardiovascular diseases, and other complications. Additionally, the synergistic effect of probiotics combined with SLE therapeutic drugs was observed in a lupus mouse model. Therefore, the use of probiotics as an adjunct of drugs might become a promising therapeutic approach in clinical practice.

Research on the effect of *L. reuteri* in SLE has been limited, and showed contradictory results. Pretreatment with *L. reuteri* DSM 17509 prolonged the remission period of lupus, delayed its onset, increased the survival rate of NZB/WF1 mice, while it increased the production of IL-10 by DCs in lupus mice (60) (**Table 1**), indicating the preventive and therapeutic effects of *L. reuteri* in SLE. *L. reuteri* was also demonstrated to exert its protective effect on lupus-associated cardiovascular disease. Administration of heat-inactivated *L. reuteri* (h-*L. reuteri*) GMNL-263 prevented cardiac remodeling and inhibited the apoptosis of myocardial cells in NZB/WF1 mice, which were characterized by a decrease in the number of TUNEL-positive cells, related components of the Fas death receptor, and number of apoptotic cells in their heart tissues (116) (**Table 1**). Moreover, supplementation with *L. reuteri* GMNL-89 or *L. reuteri* GMNL-263 alleviated liver inflammation and inhibited hepatocyte apoptosis in NZB/WF1 mice, as indicated by the improvement in liver inflammation and apoptosis indicators. Lower levels of liver proinflammatory cytokines IL-1 β, IL-6, and TNF-α were linked to the inhibition of the MAPK and NF-κB signaling pathways by *L. reuteri* (117) (**Table 1**). However, Zegara-Ruiz et al. put forward an opposing opinion in 2019. They used 2 models, one of a spontaneous lupus TLR7.1 Tg C57BL/6 mouse model, and the other of an induced lupus model using the TLR agonist imiquimod (IMQ); both of them were TLR7-dependent lupus models (67). They found that the abundance of *L. reuteri* was increased in the intestines of these 2 model mice, while the intestinal mucosal barrier was damaged, allowing the translocation of *L. reuteri* outside of the intestine. They also found that in specific pathogen-free (SPF) and germ-free (GF) lupus mice, *L. reuteri* stimulated the autoimmune reaction, which led to the damage of intestinal barrier integrity, anemia, renal tissue pathological damage, and multisystem involvement. The key features in these processes were the accumulation of plasmacytoid dendritic cells (pDCs) and the increased levels of type I IFN (67, 115) (**Table 1**). First, comparing this with the previous studies, it is obvious that different animal models were used. Second, the pathogenic effect of *L. reuteri* in the latter study was under SPF or GF conditions, and thus is not comparable to the effect of *L. reuteri* in the real complex intestinal environment. Such genetic and environmental differences affect the host-microorganism crosstalk, which have a complex relationship from the onset of the disease. Finally, the authors did not clarify the strain of *L. reuteri* used in the last study. As mentioned, differences in treatment outcomes might be caused by the different characteristics of *L. reuteri* strains. In the future, large sample studies based on a unified SLE model need to be conducted to clarify the role of *L. reuteri* in the pathogenesis of SLE.

### 3.5 Rheumatoid arthritis

Immune dysfunction and tissue inflammation are important features of RA (203, 204). The pathogenesis of RA has been correlated to imbalances in the content of oral and intestinal microorganisms. *Porphyromonas gingivalis* (*P. gingivalis*) is the pathogenic bacteria causing periodontitis. The pathological and clinical symptoms of periodontitis and RA are similar to some extent, and the risk of RA in patients with periodontitis was found to be markedly increased (205). A significant decrease in microbial diversity was observed in the non-diseased areas of periodontitis in patients at high risk of RA (206). In addition, the oral microbial composition of RA high-risk patients and patients with early RA was found to be similar, with the proportion of *Prevotella* being significantly higher than that in healthy people (207, 208). These findings suggest that the change in the composition of the microbial community might have preceded the occurrence of RA. The involvement of intestinal microbiota in the occurrence and progression of RA has also been confirmed in an animal experiment. This study used broad-spectrum antibiotics to deplete the microorganisms of collagen-induced arthritis (CIA) model mice, and found that the severity of arthritis was significantly improved and the production of intestinal proinflammatory cytokines was delayed, whereas the serum levels of proinflammatory cytokines and anti-CII antibodies were significantly decreased (209). Notably, the activity of RA was also correlated to the microbial composition (210). Although changes in the composition of oral and intestinal microorganisms might be involved in the pathogenesis of RA, their causal relationship and whether these changes are related to the production of autoantibodies remain unclear. Nevertheless, the association between microbial ecological imbalance and disease severity observed in RA animals and clinical trials has provides us with a new direction for microbial interventions in RA.

Probiotics have the ability to reduce the level of inflammation and oxidative stress in patients with RA. In a recent study, 21 patients received a probiotics mixture every day for 2 consecutive months. The level of inflammation, white blood cell count, levels of proinflammatory cytokines TNF-α and IL-6, and levels of NOx were decreased, whereas the level of sulfhydryl (SH) and total radiotrapping antioxidant parameters (TRAP) were increased in the intervention group, indicating a decrease in the level of oxidative stress and an increase in the level of antioxidant parameters (211). This suggests that probiotics regulate the levels of inflammatory and oxidative stress indicators. Further studies should be conducted on the relationship between the probiotic-induced improvement of these indicators and the incidence rate, clinical symptoms, and prognosis of RA. A healthy diet might have a protective effect on RA (212, 213). Based on this, researchers combined probiotics with food ingredients with anti-inflammatory and antioxidant properties to explore their combined efficacy against RA. For example, a study investigated the effect of an anti-inflammatory diet containing probiotics and some food ingredients with anti-inflammatory effects in patients with RA and found that the 28 joints-Erythrocyte Seduction Rate (DAS28-ESR) and levels of 28 joints-C-reactive protein (DAS28-CRP), which reflect the activity of RA, were significantly reduced and the proportion of joint tenderness relief was higher than that of the control group (115). In another study, 50 patients with RA consumed an anti-inflammatory diet supplemented with probiotics. After 10 weeks, the levels of triglycerides (TG), high density lipoprotein cholesterol (HDL-C), apolipoprotein-B100/A1, fatty acids, and other blood lipid indicators of patients exhibited a trend towards the direction of improved prognosis (214). Based on these two studies, and despite the inability to determine whether the decline in RA activity and improvement of blood lipid status were the result of specific dietary ingredients or probiotics, it was concluded that the combination of probiotics and anti-inflammatory foods might have synergistic effects, providing potential reference significance for formulating a daily dietary strategy for patients with RA.

As *L. reuteri* regulates immune function through a variety of mechanisms, researchers have attempted to use it for the treatment of RA. In one study, where mice were administered h-*L. reuteri* MM2-3 before and after CIA induction, it was observed that h-*L. reuteri* pretreatment reduced the severity and incidence of CIA in mice. In addition, they also observed that the serum levels of type II collagen-specific immunoglobulin G (CII-IgG) and proinflammatory cytokines IL-6 and CXCL1 were decreased, whereas those of the anti-inflammatory cytokine IL-10 were increased. The improvement of CIA by h-*L. reuteri* pretreatment might be related to the reduction in the level of IL-6 and stimulation of production of CD103^+^ DCs, which, in turn, promote the production and peripheral migration of Tregs. However, it is noteworthy that although CIA did not occur in mice pretreated with h-*L. reuteri*, pretreatment had no effect on mice with developing CIA (53) (**Table 1**). Another study found that pretreatment with a probiotic mixture containing *L. reuteri* CCFM 8631 and *L. reuteri* CCFM 14 before the occurrence of CIA reduced the swelling and pathological damage in the joints of rats, suggesting its potential use for the prevention of CIA. In addition to the changes in the levels of proinflammatory cytokines and collagen-specific antibodies, this study also observed the levels of Th1-secreted cytokine IFN-γ and IL-12, which promotes Th1 cell differentiation, were decreased. In addition, the proportion of SCFA-producing microbial communities was increased. Notably, the authors found that the levels of the anti-inflammatory cytokine IL-10 were significantly decreased after treatment with *L. reuteri*, which was in contradiction with the changes in the levels of IL-10 observed in the former study. They hence assumed that IL-10 secreted by Tregs exerts anti-inflammatory and immunosuppressive effects, whereas IL-10 secreted by non-T-cells might aggravate the production of autoantibodies in patients with RA. In this study, the high levels of IL-10 in CIA rats were mainly produced by non-T-cells, with *L. reuteri* playing a protective role by regulating the number of non-T-cells and reducing the levels of destructive IL-10 (118) (**Table 1**). Recent research has suggested that *L. reuteri* can prevent CIA; however, the treatment effect on CIA remains unclear. LrS235, a genetically engineered *L. reuteri* secreting Kv1.3 potassium blocker ShK-235, significantly reduced clinical symptoms and joint inflammation in RA rats. This may be related to functional ShK-235 inhibiting T effector memory (TEM) cells (215). Although the functional substances produced by such bioengineering benefit the body, we should also pay attention to its immunogenicity. As RA is a chronic disease, the management of daily lifestyle and diet cannot be ignored. Based on recent developments in this field, adding *L. reuteri* to the daily diet might be a new strategy for the prevention of RA in the future. However, these findings need to be further explored and verified in the clinical setting.

### 3.6 Multiple sclerosis

Multiple sclerosis (MS) is an autoimmune disease characterized by central nervous system (CNS) demyelination, axonal injury, and neurodegeneration as the main pathological changes (216, 217). The participation of intestinal microbiota in the pathogenesis of MS has been preliminarily verified in an animal model of experimental autoimmune encephalopathy (EAE). The diversity of intestinal microflora, number of *Lactobacillus*, *Bacteroides*, and *Prevotellaceae*, and antioxidant level of intestinal microflora were increased in EAE mice fed an intermittent diet. Notably, transplantation of their fecal matter to EAE mice fed a normal diet resulted in the relief of symptoms (218). Some microorganisms that mediate the intensification or protection of EAE have also been observed in human intestines. For example, an increase in the abundance of *Anaerotruncus colihomis* was reported to improve EAE, and has been demonstrated to exert potential beneficial effects on patients with MS, such as reducing the incidence of disability (219, 220). The clinical classification of MS includes relapsing remitting (RR), secondary progressive (SP), primary progressive (PP), and progressive relapsing (PR) disease. However, these different phenotypes do not correlate with increasing imbalances in the composition of microorganisms (221, 222). For instance, compared with RRMS, the proportion of *Enteroberiaceae* and *Clostridium* in progressive MS was increased, whereas that of *Blautia* and *Agathobaculum* was decreased. *Clostridium* has been associated with higher disability and fatigue rates (220). The microflora of different types of MS have characteristic community characteristics, and can thus serve as biomarkers for determining the stage and severity of the disease (221). Clarifying the microbial composition of several types of MS might have great guiding significance for early clinical diagnosis and intervention. Specific microorganism might also serve as indicators of MS activity. Studies have shown that the presence of two of five microorganisms, namely *Odoribacter* and *Butyricicoccus*, has been significantly related to the risk of poor imaging performance of brain MRI (223). The pathogenesis of microbial-mediated MS might be related to the low level of butyrate and indolelactate (224), which is the intermediate product of indole propionate in Try metabolism (225). A decrease in the number of indolelactate-producing microbiota might lead to a decrease in the serum levels of indolelactate and indolepropionate (224). Indolepropionate is considered to be an antioxidant with significant neuroprotective effects (226). Accordingly, the inflammatory state in the body might be related to the decline in the serum levels of these metabolites (224). In general, we should focus not only on the microorganism itself that might serve as a biomarker and intervention target of MS pathogenicity, but also in its metabolites.

The outcomes of clinical trials on the use of probiotics in patients with MS have been positive. A study found that oral administration of 3600 billion CFU/d of a probiotics mixture containing 8 strains for 2 consecutive months not only reduced the number of intermediate monocytes (CD14^high^CD16^low^), but also decreased the expression of human leukocyte antigen DR (HLA-DR) in DCs of patients with RRMS. In addition, a reduction in the number of inflammatory monocytes (CD14^low^ CD16^high^) and expression of costimulatory marker CD80 of classical monocytes was observed in the healthy control group. Conversely, after discontinuation of the use of probiotics, the number of inflammatory monocytes was increased, whereas that of Tregs was decreased (227). This showed that the probiotics mixture regulated the innate immune cells of patients with MS and healthy controls, and promoted anti-inflammatory reactions *in vivo* (227). Another study found that the serum levels of the proinflammatory cytokine IL-6 were decreased, whereas that of the brain-derived neurotrophic factor (BDNF) were significantly increased after the use of multiple probiotics (228). BDNF is a neuroregulatory protein that plays an important role in maintaining the survival and differentiation of neurons and can improve psychological symptoms, such as depression and anxiety (229). By using the mental health questionnaire to determine the depression score, fatigue degree, and pain assessment, the mental health of patients with MS was shown to be improved after the use of probiotics (228). Results from current clinical trials have indicated that probiotics might be used as potential candidates for the treatment of MS. In the future, more trials should be carried out on patients with different MS types on the basis of acquiring unified inclusion criteria to verify whether the use of probiotics can really benefit patients with MS.

Trp metabolites combined with AHR have been reported to exert immunoregulatory and anti-inflammatory effects and enhance gastrointestinal barrier function (225). Ampicillin (Amp)-sensitive vancomycin (Vanco)-resistant bacteria catalyze the binding of Trp to AHR ligands (34). *L. reuteri* is an Amp-sensitive Vanco-resistant bacterium, which metabolizes Trp to AHR agonists, acts on AHR of astrocytes, reduces inflammation of the central nervous system, and plays a protective role on EAE (119) (**Table 1**). Further supporting evidence have suggested that *L. reuteri* DSM 17938 improved the immune response of EAE by regulating the relative abundance of “beneficial” bacteria, such as *Bifidobacterium*, *Prevotella*, and *Lactobacillus* and that of harmful bacteria, such as *Anaeroplasma*, *Rikenellaceae*, and *Clostridium*, and restoring the low diversity of intestinal microorganisms. In another study, the numbers of Th1 and Th17 cells and levels of their cytokines were decreased in EAE mice (120) (**Table 1**). With respect to the role of *L. reuteri* in EAE, Miyauchi et al. reached a diametrically opposite conclusion in their subsequent study. They assumed that *L. reuteri* as a host symbiotic bacteria would aggravate the disease of genetically susceptible EAE mice and change their systemic metabolism. They accordingly found that *L. reuteri* and Erysipelaceae bacteria co-colonized and synergistically induced pathogenicity in EAE mice. This might be due to the similarity of *L. reuteri* to the myelin oligodendrocyte glycoprotein (MOG), which activates the MOG-specific CD4^+^ T-cells in the small intestine, causing an attack by autoimmune T-cells and worsening of spinal inflammation (58) (**Table 1**). *L. reuteri* is considered to be a strain that can interact with the genetically susceptible host to aggravate the symptoms of EAE (230). However, this contradictory conclusion might be related to difference among *L. reuteri* strains. Concomitantly, we should also note that *L. reuteri* can exert a synergistic effect with other microorganisms, potentially either benefiting health or causing disease. Therefore, we cannot ignore the “assistant” role of *L. reuteri*. Further research on MS-related intestinal microorganisms might help to identify more relevant species. At present, *L. reuteri* has only been applied to an animal model of MS, and has not been tested in clinical trials. Whether it can improve EAE remains undetermined and requires further research in clinical trials. Hence, there is still a long way to go before it becomes a new candidate for the future prevention and treatment of MS.

## 4 Conclusion

With the development of multiomics, the integration of metagenomics, macrotranscriptomics, metabolomics, and immunological data provides great assistance to the study of the mechanism of microbe-host crosstalk. The ability of probiotics to adhere to and colonize the gut may underlie a variety of mechanisms. In the complex immune regulation mechanism, *L. reuteri* and its metabolites play a central role in protecting intestinal barrier function and regulating immune cells. Multiple results have shown that *L. reuteri* affects the number and differentiation of DCs, which in turn affects the differentiation of T cells and the proportion of its subsets, playing a role in the regulation of the host immune system. However, due to the influence of host genetics, epigenetics and environment, especially diet, Probiotics have differentiated immune regulation in individuals. These insights highlight the importance of studying the level of probiotic strains and differences in individual physiological characteristics. The analysis of the molecular structure of probiotics and host cell surface receptors will help us to further understand its mechanism of action, and more meaningful conclusions will be achieved in the future by unifying experimental models and standardized samples.

The results of some animal and clinical studies indicated the encouraging application prospect of *L. reuteri*. It is undeniable that the research on probiotics had obvious heterogeneity, including the animals or people included in the study, the choice of the control group, the type and combination of probiotics selected, the intervention dose, the duration of the intervention or experiment, the environment and diets. These are reasons for contradictory or ambiguous conclusions. In addition, the efficacy of probiotics were evaluated based on clinical data, which were collected and analyzed differently. For example, many reports had obvious subjectivity in the evaluation of emotional or social function. In the future, we need to adopt high-quality research methods and carry out research on the basis of unified evaluation criteria.

With the in-depth study of probiotics, more details of the application of L. reuteri have been derived. No study has explored its metabolism *in vivo*, which is closely related to its safety; The biofilm state of *L. reuteri* optimizes the traditional form of drug delivery, but also requires close monitoring and control to avoid the excessive proliferation of *L. reuteri*; At present, there are two kinds of understanding on the choice of single strain or multi-strain combination, one is that microbiota have a complex network of relationships, with interconnected components that interact with each other to jointly maintain the microecological balance. Therefore, the synergistic effect of multiple strains may be more conducive to the inflammatory response and the stability of the immune system. The other believes that a single strain exhibiting a definite effect was more effective. This is because mixed strains might get out of control due to the inconsistent reproduction speed of each strain, thus disturbing the balance and hindering the control of microecology.

Generally speaking, we need to study further the metabolic pathway of *L. reuteri* in human body, determine the precise efficacy of different strains, comprehensively consider the safety and efficacy of the strain, carefully select the best strain or its combination, adopt the appropriate dose at the right time and maintain it for enough time. This may provide guidance for the clinical application of *L. reuteri* in the future.

## Author contributions

ZL and AC designed and wrote the manuscript. RY is responsible for the concept development, funding acquisition, review and revision. AX, XL and SJ searched the literature. All authors approved the manuscript for public. All authors contributed to the article and approved the submitted version.

## References

[1] Kim SK Guevarra RB Kim YT Kwon J Kim H Cho JH. Role of probiotics in human gut microbiome-associated diseases. J Microbiol Biotechnol (2019) 29(9):1335–40. doi: 10.4014/jmb.1906.06064 31434172

[2] Wieers G Belkhir L Enaud R Leclercq S Philippart de Foy JM Dequenne I. How probiotics affect the microbiota. Front Cell Infect Microbiol (2019) 9:454. doi: 10.3389/fcimb.2019.00454 32010640 PMC6974441

[3] Jardou M Brossier C Guiyedi K Faucher Q Lawson R. Pharmacological hypothesis: A recombinant probiotic for taming bacterial beta-glucuronidase in drug-induced enteropathy. Pharmacol Res Perspect (2022) 10(5):e00998. doi: 10.1002/prp2.998 36082825 PMC9460963

[4] Pique N Berlanga M Minana-Galbis D. Health benefits of heat-killed (Tyndallized) probiotics: an overview. Int J Mol Sci (2019) 20(10):2534. doi: 10.3390/ijms20102534 31126033 PMC6566317

[5] Krawczyk RT Banaszkiewicz A. Dr. Jozef Brudzinski - the true 'Father of probiotics'. Benef Microbes (2021) 12(3):211–3. doi: 10.3920/BM2020.0201 34057052

[6] Gangaiah D Mane SP Tawari NR Lakshmanan N Ryan V Volland A. In silico, in *vitro* and in *vivo* safety evaluation of Limosilactobacillus reuteri strains ATCC PTA-126787 & ATCC PTA-126788 for potential probiotic applications. PloS One (2022) 17(1):e0262663. doi: 10.1371/journal.pone.0262663 35081129 PMC8791467

[7] Anjum J Nazir S Tariq M Barrett K Zaidi A. Lactobacillus commensals autochthonous to human milk have the hallmarks of potent probiotics. Microbiol (Reading). (2020) 166(10):966–80. doi: 10.1099/mic.0.000966 32886600

[8] Bindu A Lakshmidevi N. Identification and in *vitro* evaluation of probiotic attributes of lactic acid bacteria isolated from fermented food sources. Arch Microbiol (2021) 203(2):579–95. doi: 10.1007/s00203-020-02037-0 32990771

[9] Jomehzadeh N Javaherizadeh H Amin M Saki M Al-Ouqaili MTS Hamidi H. Isolation and identification of potential probiotic Lactobacillus species from feces of infants in southwest Iran. Int J Infect Dis (2020) 96:524–30. doi: 10.1016/j.ijid.2020.05.034 32439543

[10] Bull M Plummer S Marchesi J Mahenthiralingam E. The life history of Lactobacillus acidophilus as a probiotic: a tale of revisionary taxonomy, misidentification and commercial success. FEMS Microbiol Lett (2013) 349(2):77–87. doi: 10.1111/1574-6968.12293 24152174

[11] Kubota M Ito K Tomimoto K Kanazaki M Tsukiyama K Kubota A. Lactobacillus reuteri DSM 17938 and magnesium oxide in children with functional chronic constipation: A double-blind and randomized clinical trial. Nutrients (2020) 12(1):225. doi: 10.3390/nu12010225 31952280 PMC7019518

[12] Korpela K Salonen A Vepsalainen O Suomalainen M Kolmeder C Varjosalo M. Probiotic supplementation restores normal microbiota composition and function in antibiotic-treated and in caesarean-born infants. Microbiome (2018) 6(1):182. doi: 10.1186/s40168-018-0567-4 30326954 PMC6192119

[13] Slattery C Cotter PD O'Toole PW. Analysis of health benefits conferred by lactobacillus species from Kefir. Nutrients (2019) 11(6):1252. doi: 10.3390/nu11061252 31159409 PMC6627492

[14] Rao NS Lundberg L Palmkron S Hakansson S Bergenstahl B Carlquist M. Flow cytometric analysis reveals culture condition dependent variations in phenotypic heterogeneity of Limosilactobacillus reuteri. Sci Rep (2021) 11(1):23567. doi: 10.1038/s41598-021-02919-3 34876641 PMC8651721

[15] Wegmann U MacKenzie DA Zheng J Goesmann A Roos S Swarbreck D. The pan-genome of Lactobacillus reuteri strains originating from the pig gastrointestinal tract. BMC Genomics (2015) 16:1023. doi: 10.1186/s12864-015-2216-7 26626322 PMC4667477

[16] Shokryazdan P Faseleh Jahromi M Liang JB Ho YW. Probiotics: from isolation to application. J Am Coll Nutr (2017) 36(8):666–76. doi: 10.1080/07315724.2017.1337529 28937854

[17] Espirito Santo C Caseiro C Martins MJ Monteiro R Brandao I. Gut microbiota, in the halfway between nutrition and lung function. Nutrients (2021) 13(5):1716. doi: 10.3390/nu13051716 34069415 PMC8159117

[18] Liu AH. Revisiting the hygiene hypothesis for allergy and asthma. J Allergy Clin Immunol (2015) 136(4):860–5. doi: 10.1016/j.jaci.2015.08.012 26449798

[19] MacKenzie DA Jeffers F Parker ML Vibert-Vallet A Bongaerts RJ Roos S. Strain-specific diversity of mucus-binding proteins in the adhesion and aggregation properties of Lactobacillus reuteri. Microbiol (Reading) (2010) 156(Pt 11):3368–78. doi: 10.1099/mic.0.043265-0 20847011

[20] Liu M Hu R Guo Y Sun W Li J Fan M. [Influence of Lactobacillus reuteri SL001 on intestinal microbiota in AD model mice and C57BL/6 mice]. Sheng Wu Gong Cheng Xue Bao. (2020) 36(9):1887–98. doi: 10.13345/j.cjb.200024 33164464

[21] Garg S Singh TP Malik RK. *In vivo* implications of potential probiotic lactobacillus reuteri LR6 on the gut and immunological parameters as an adjuvant against protein energy malnutrition. Probiotics Antimicrob Proteins. (2020) 12(2):517–34. doi: 10.1007/s12602-019-09563-4 31218544

[22] Yang J Wang C Liu L Zhang M. Lactobacillus reuteri KT260178 supplementation reduced morbidity of piglets through its targeted colonization, improvement of cecal microbiota profile, and immune functions. Probiotics Antimicrob Proteins. (2020) 12(1):194–203. doi: 10.1007/s12602-019-9514-3 30659502

[23] Liu Y Tian X He B Hoang TK Taylor CM Blanchard E. Lactobacillus reuteri DSM 17938 feeding of healthy newborn mice regulates immune responses while modulating gut microbiota and boosting beneficial metabolites. Am J Physiol Gastrointest Liver Physiol (2019) 317(6):G824–G38. doi: 10.1152/ajpgi.00107.2019 PMC6962498 31482733

[24] Hou C Liu H Zhang J Zhang S Yang F Zeng X. Intestinal microbiota succession and immunomodulatory consequences after introduction of Lactobacillus reuteri I5007 in neonatal piglets. PloS One (2015) 10(3):e0119505. doi: 10.1371/journal.pone.0119505 25775260 PMC4361599

[25] Wasfi R Abd El-Rahman OA Zafer MM Ashour HM. Probiotic Lactobacillus sp. inhibit growth, biofilm formation and gene expression of caries-inducing Streptococcus mutans. J Cell Mol Med (2018) 22(3):1972–83. doi: 10.1111/jcmm.13496 PMC5824418 29316223

[26] Talarico TL Dobrogosz WJ. Chemical characterization of an antimicrobial substance produced by Lactobacillus reuteri. Antimicrob Agents Chemother (1989) 33(5):674–9. doi: 10.1128/AAC.33.5.674 PMC172512 2751282

[27] Talarico TL Dobrogosz WJ. Purification and characterization of glycerol dehydratase from lactobacillus reuteri. Appl Environ Microbiol (1990) 56(4):1195–7. doi: 10.1128/aem.56.4.1195-1197.1990 PMC184372 16348166

[28] Chen G Chen J. A novel cell modification method used in biotransformation of glycerol to 3-HPA by Lactobacillus reuteri. Appl Microbiol Biotechnol (2013) 97(10):4325–32. doi: 10.1007/s00253-013-4723-2 23359000

[29] Stevens JF Maier CS. Acrolein: sources, metabolism, and biomolecular interactions relevant to human health and disease. Mol Nutr Food Res (2008) 52(1):7–25. doi: 10.1002/mnfr.200700412 18203133 PMC2423340

[30] Engels C Schwab C Zhang J Stevens MJ Bieri C Ebert MO. Acrolein contributes strongly to antimicrobial and heterocyclic amine transformation activities of reuterin. Sci Rep (2016) 6:36246. doi: 10.1038/srep36246 27819285 PMC5098142

[31] Wu H Xie S Miao J Li Y Wang Z Wang M. Lactobacillus reuteri maintains intestinal epithelial regeneration and repairs damaged intestinal mucosa. Gut Microbes (2020) 11(4):997–1014. doi: 10.1080/19490976.2020.1734423 32138622 PMC7524370

[32] Nation ML Dunne EM Joseph SJ Mensah FK Sung V Satzke C. Impact of Lactobacillus reuteri colonization on gut microbiota, inflammation, and crying time in infant colic. Sci Rep (2017) 7(1):15047. doi: 10.1038/s41598-017-15404-7 29118383 PMC5678104

[33] Husain S Allotey J Drymoussi Z Wilks M Fernandez-Felix BM Whiley A. Effects of oral probiotic supplements on vaginal microbiota during pregnancy: a randomised, double-blind, placebo-controlled trial with microbiome analysis. BJOG (2020) 127(2):275–84. doi: 10.1111/1471-0528.15675 PMC6973149 30932317

[34] Zelante T Iannitti RG Cunha C De Luca A Giovannini G Pieraccini G. Tryptophan catabolites from microbiota engage aryl hydrocarbon receptor and balance mucosal reactivity via interleukin-22. Immunity (2013) 39(2):372–85. doi: 10.1016/j.immuni.2013.08.003 23973224

[35] Spits H Artis D Colonna M Diefenbach A Di Santo JP Eberl G. Innate lymphoid cells–a proposal for uniform nomenclature. Nat Rev Immunol (2013) 13(2):145–9. doi: 10.1038/nri3365 23348417

[36] Cervantes-Barragan L Chai JN Tianero MD Di Luccia B Ahern PP Merriman J. Lactobacillus reuteri induces gut intraepithelial CD4(+)CD8alphaalpha(+) T cells. Science (2017) 357(6353):806–10. doi: 10.1126/science.aah5825 PMC5687812 28775213

[37] Yang KM Kim JS Kim HS Kim YY Oh JK Jung HW. Lactobacillus reuteri AN417 cell-free culture supernatant as a novel antibacterial agent targeting oral pathogenic bacteria. Sci Rep (2021) 11(1):1631. doi: 10.1038/s41598-020-80921-x 33452304 PMC7810884

[38] Yang F Wang A Zeng X Hou C Liu H Qiao S. Lactobacillus reuteri I5007 modulates tight junction protein expression in IPEC-J2 cells with LPS stimulation and in newborn piglets under normal conditions. BMC Microbiol (2015) 15:32. doi: 10.1186/s12866-015-0372-1 25888437 PMC4350629

[39] Yuki T Yoshida H Akazawa Y Komiya A Sugiyama Y Inoue S. Activation of TLR2 enhances tight junction barrier in epidermal keratinocytes. J Immunol (Baltimore Md 1950). (2011) 187(6):3230–7. doi: 10.4049/jimmunol.1100058 21841130

[40] Martinez RC Seney SL Summers KL Nomizo A De Martinis EC Reid G. Effect of Lactobacillus rhamnosus GR-1 and Lactobacillus reuteri RC-14 on the ability of Candida albicans to infect cells and induce inflammation. Microbiol Immunol (2009) 53(9):487–95. doi: 10.1111/j.1348-0421.2009.00154.x 19703242

[41] Xie S Zhao S Jiang L Lu L Yang Q Yu Q. Lactobacillus reuteri Stimulates Intestinal Epithelial Proliferation and Induces Differentiation into Goblet Cells in Young Chickens. J Agric Food Chem (2019) 67(49):13758–66. doi: 10.1021/acs.jafc.9b06256 31789514

[42] Wang Q Sun Q Wang J Qiu X Qi R Huang J. Identification of differentially expressed miRNAs after Lactobacillus reuteri treatment in the ileum mucosa of piglets. Genes Genomics (2020) 42(11):1327–38. doi: 10.1007/s13258-020-00998-6 32980994

[43] Khmaladze I Butler E Fabre S Gillbro JM. Lactobacillus reuteri DSM 17938-A comparative study on the effect of probiotics and lysates on human skin. Exp Dermatol (2019) 28(7):822–8. doi: 10.1111/exd.13950 31021014

[44] Park ES Freeborn J Venna VR Roos S Rhoads JM Liu Y. Lactobacillus reuteri effects on maternal separation stress in newborn mice. Pediatr Res (2021) 90(5):980–8. doi: 10.1038/s41390-021-01374-0 33531679

[45] Gupta N Ferreira J Hong CHL Tan KS. Lactobacillus reuteri DSM 17938 and ATCC PTA 5289 ameliorates chemotherapy-induced oral mucositis. Sci Rep (2020) 10(1):16189. doi: 10.1038/s41598-020-73292-w 33004948 PMC7530769

[46] Xie W Song L Wang X Xu Y Liu Z Zhao D. A bovine lactoferricin-lactoferrampin-encoding Lactobacillus reuteri CO21 regulates the intestinal mucosal immunity and enhances the protection of piglets against enterotoxigenic Escherichia coli K88 challenge. Gut Microbes (2021) 13(1):1956281. doi: 10.1080/19490976.2021.1956281 34369287 PMC8354667

[47] Tang J Guo C Gong F. [Protective effect of Lactobacillus reuteri against oxidative stress in neonatal mice with necrotizing enterocolitis]. Nan Fang Yi Ke Da Xue Xue Bao. (2019) 39(10):1221–6. doi: 10.12122/j.issn.1673-4254.2019.10.14 PMC6867940 31801706

[48] Karimi S Jonsson H Lundh T Roos S. Lactobacillus reuteri strains protect epithelial barrier integrity of IPEC-J2 monolayers from the detrimental effect of enterotoxigenic Escherichia coli. Physiol Rep (2018) 6(2):e13514. doi: 10.14814/phy2.13514 29368445 PMC5789714

[49] Hoang TK He B Wang T Tran DQ Rhoads JM Liu Y. Protective effect of Lactobacillus reuteri DSM 17938 against experimental necrotizing enterocolitis is mediated by Toll-like receptor 2. Am J Physiol Gastrointest Liver Physiol (2018) 315(2):G231–G40. doi: 10.1152/ajpgi.00084.2017 PMC6139641 29648878

[50] Zhao Y Qi C Li X Lu M Zhang H Zhou J. Prevention of atopic dermatitis in mice by lactobacillus reuteri Fn041 through induction of regulatory T cells and modulation of the gut microbiota. Mol Nutr Food Res (2022) 66(6):e2100699. doi: 10.1002/mnfr.202100699 34825773

[51] Liu Y Tran DQ Fatheree NY Marc Rhoads J. Lactobacillus reuteri DSM 17938 differentially modulates effector memory T cells and Foxp3+ regulatory T cells in a mouse model of necrotizing enterocolitis. Am J Physiol Gastrointest Liver Physiol (2014) 307(2):G177–86. doi: 10.1152/ajpgi.00038.2014 PMC4101683 24852566

[52] Savino F Galliano I Garro M Savino A Dapra V Montanari P. Regulatory T cells and Toll-like receptor 2 and 4 mRNA expression in infants with colic treated with Lactobacillus reuteri DSM17938. Benef Microbes (2018) 9(6):917–25. doi: 10.3920/BM2017.0194 30406696

[53] Jia H Ren S Wang X. Heat-killed probiotic regulates the body's regulatory immunity to attenuate subsequent experimental autoimmune arthritis. Immunol Lett (2019) 216:89–96. doi: 10.1016/j.imlet.2019.10.009 31644891

[54] Ahlberg E Marti M Govindaraj D Severin E Duchen K Jenmalm MC. Immune-related microRNAs in breast milk and their relation to regulatory T cells in breastfed children. Pediatr Allergy Immunol (2023) 34(4):e13952. doi: 10.1111/pai.13952 37102392

[55] Forsberg A HuOman J Soderholm S Bhai Mehta R Nilsson L Abrahamsson TR. Pre- and postnatal Lactobacillus reuteri treatment alters DNA methylation of infant T helper cells. Pediatr Allergy Immunol (2020) 31(5):544–53. doi: 10.1111/pai.13240 32150651

[56] Valeur N Engel P Carbajal N Connolly E Ladefoged K. Colonization and immunomodulation by Lactobacillus reuteri ATCC 55730 in the human gastrointestinal tract. Appl Environ Microbiol (2004) 70(2):1176–81. doi: 10.1128/AEM.70.2.1176-1181.2004 PMC348788 14766603

[57] Ferreira RC Forsyth LE Richman PI Wells C Spencer J MacDonald TT. Changes in the rate of crypt epithelial cell proliferation and mucosal morphology induced by a T-cell-mediated response in human small intestine. Gastroenterology (1990) 98(5 Pt 1):1255–63. doi: 10.1016/0016-5085(90)90342-X 2138987

[58] Miyauchi E Kim SW Suda W Kawasumi M Onawa S Taguchi-Atarashi N. Gut microorganisms act together to exacerbate inflammation in spinal cords. Nature (2020) 585(7823):102–6. doi: 10.1038/s41586-020-2634-9 32848245

[59] Engevik MA Ruan W Esparza M Fultz R Shi Z Engevik KA. Immunomodulation of dendritic cells by Lactobacillus reuteri surface components and metabolites. Physiol Rep (2021) 9(2):e14719. doi: 10.14814/phy2.14719 33463911 PMC7814497

[60] Manirarora JN Kosiewicz MM Alard P. Feeding lactobacilli impacts lupus progression in (NZBxNZW)F1 lupus-prone mice by enhancing immunoregulation. Autoimmunity (2020) 53(6):323–32. doi: 10.1080/08916934.2020.1777282 32552071

[61] Liu HY Gu F Zhu C Yuan L Zhu C Zhu M. Epithelial heat shock proteins mediate the protective effects of limosilactobacillus reuteri in dextran sulfate sodium-induced colitis. Front Immunol (2022) 13:865982. doi: 10.3389/fimmu.2022.865982 35320932 PMC8934773

[62] Coombes JL Siddiqui KR Arancibia-Carcamo CV Hall J Sun CM Belkaid Y. A functionally specialized population of mucosal CD103+ DCs induces Foxp3+ regulatory T cells via a TGF-beta and retinoic acid-dependent mechanism. J Exp Med (2007) 204(8):1757–64. doi: 10.1084/jem.20070590 PMC2118683 17620361

[63] Eloranta ML Ronnblom L. Cause and consequences of the activated type I interferon system in SLE. J Mol Med (Berl). (2016) 94(10):1103–10. doi: 10.1007/s00109-016-1421-4 PMC5052287 27094810

[64] Kim JM Park SH Kim HY Kwok SK. A plasmacytoid dendritic cells-type I interferon axis is critically implicated in the pathogenesis of systemic lupus erythematosus. Int J Mol Sci (2015) 16(6):14158–70. doi: 10.3390/ijms160614158 PMC4490545 26110387

[65] Davison LM Jorgensen TN. New treatments for systemic lupus erythematosus on the horizon: targeting plasmacytoid dendritic cells to inhibit cytokine production. J Clin Cell Immunol (2017) 8(6):534. doi: 10.4172/2155-9899.1000534 29430334 PMC5804747

[66] Crow MK. Type I interferon in the pathogenesis of lupus. J Immunol (Baltimore Md 1950). (2014) 192(12):5459–68. doi: 10.4049/jimmunol.1002795 PMC4083591 24907379

[67] Zegarra-Ruiz DF El Beidaq A Iniguez AJ Lubrano Di Ricco M Manfredo Vieira S Ruff WE. A diet-sensitive commensal lactobacillus strain mediates TLR7-dependent systemic autoimmunity. Cell Host Microbe (2019) 25(1):113–27 e6. doi: 10.1016/j.chom.2018.11.009 30581114 PMC6377154

[68] Jarade A Di Santo JP Serafini N. Group 3 innate lymphoid cells mediate host defense against attaching and effacing pathogens. Curr Opin Microbiol (2021) 63:83–91. doi: 10.1016/j.mib.2021.06.005 34274597

[69] Wen Z Chen D Bian J. [Role of group 3 innate lymphoid cells in intestinal barrier]. Zhonghua Wei Zhong Bing Ji Jiu Yi Xue. (2019) 31(2):252–6. doi: 10.3760/cma.j.issn.2095-4352.2019.02.028 30827322

[70] Song D Lai L Ran Z. Metabolic regulation of group 3 innate lymphoid cells and their role in inflammatory bowel disease. Front Immunol (2020) 11:580467. doi: 10.3389/fimmu.2020.580467 33193381 PMC7649203

[71] Wang T Zheng N Luo Q Jiang L He B Yuan X. Probiotics lactobacillus reuteri abrogates immune checkpoint blockade-associated colitis by inhibiting group 3 innate lymphoid cells. Front Immunol (2019) 10:1235. doi: 10.3389/fimmu.2019.01235 31214189 PMC6558076

[72] Mu Q Swartwout BK Edwards M Zhu J Lee G Eden K. Regulation of neonatal IgA production by the maternal microbiota. Proc Natl Acad Sci U.S.A. (2021) 118(9):e2015691118. doi: 10.1073/pnas.2015691118 33619092 PMC7936341

[73] Jiang P Yang W Jin Y Huang H Shi C Jiang Y. Lactobacillus reuteri protects mice against Salmonella typhimurium challenge by activating macrophages to produce nitric oxide. Microb Pathog (2019) 137:103754. doi: 10.1016/j.micpath.2019.103754 31539587

[74] De Gregorio PR Juarez Tomas MS Nader-Macias ME. Immunomodulation of lactobacillus reuteri CRL1324 on group B streptococcus vaginal colonization in a murine experimental model. Am J Reprod Immunol (New York NY 1989). (2016) 75(1):23–35. doi: 10.1111/aji.12445 26547516

[75] Cario E Gerken G Podolsky DK. Toll-like receptor 2 controls mucosal inflammation by regulating epithelial barrier function. Gastroenterology (2007) 132(4):1359–74. doi: 10.1053/j.gastro.2007.02.056 17408640

[76] Collins CB Ho J Wilson TE Wermers JD Tlaxca JL Lawrence MB. CD44 deficiency attenuates chronic murine ileitis. Gastroenterology (2008) 135(6):1993–2002. doi: 10.1053/j.gastro.2008.08.053 18854186 PMC4418802

[77] Baaten BJ Li CR Bradley LM. Multifaceted regulation of T cells by CD44. Communicative Integr Biol (2010) 3(6):508–12. doi: 10.4161/cib.3.6.13495 PMC3038050 21331226

[78] Lee WT Vitetta ES. The differential expression of homing and adhesion molecules on virgin and memory T cells in the mouse. Cell Immunol (1991) 132(1):215–22. doi: 10.1016/0008-8749(91)90020-C 1676613

[79] Kornete M Piccirillo CA. Functional crosstalk between dendritic cells and Foxp3(+) regulatory T cells in the maintenance of immune tolerance. Front Immunol (2012) 3:165. doi: 10.3389/fimmu.2012.00165 22737152 PMC3381230

[80] Gabryszewski SJ Bachar O Dyer KD Percopo CM Killoran KE Domachowske JB. Lactobacillus-mediated priming of the respiratory mucosa protects against lethal pneumovirus infection. J Immunol (Baltimore Md 1950). (2011) 186(2):1151–61. doi: 10.4049/jimmunol.1001751 PMC3404433 21169550

[81] Lin YP Thibodeaux CH Pena JA Ferry GD Versalovic J. Probiotic Lactobacillus reuteri suppress proinflammatory cytokines via c-Jun. Inflammatory bowel diseases. (2008) 14(8):1068–83. doi: 10.1002/ibd.20448 18425802

[82] Lee J Yang W Hostetler A Schultz N Suckow MA Stewart KL. Characterization of the anti-inflammatory Lactobacillus reuteri BM36301 and its probiotic benefits on aged mice. BMC Microbiol (2016) 16:69. doi: 10.1186/s12866-016-0686-7 27095067 PMC4837529

[83] Amar Y Rizzello V Cavaliere R Campana S De Pasquale C Barberi C. Divergent signaling pathways regulate IL-12 production induced by different species of Lactobacilli in human dendritic cells. Immunol Lett (2015) 166(1):6–12. doi: 10.1016/j.imlet.2015.05.001 25977118

[84] Bottcher MF Abrahamsson TR Fredriksson M Jakobsson T Bjorksten B. Low breast milk TGF-beta2 is induced by Lactobacillus reuteri supplementation and associates with reduced risk of sensitization during infancy. Pediatr Allergy Immunol (2008) 19(6):497–504. doi: 10.1111/j.1399-3038.2007.00687.x 18221472

[85] Hendrikx T Duan Y Wang Y Oh JH Alexander LM Huang W. Bacteria engineered to produce IL-22 in intestine induce expression of REG3G to reduce ethanol-induced liver disease in mice. Gut (2019) 68(8):1504–15. doi: 10.1136/gutjnl-2018-317232 PMC6387784 30448775

[86] Mackos AR Galley JD Eubank TD Easterling RS Parry NM Fox JG. Social stress-enhanced severity of Citrobacter rodentium-induced colitis is CCL2-dependent and attenuated by probiotic Lactobacillus reuteri. Mucosal Immunol (2016) 9(2):515–26. doi: 10.1038/mi.2015.81 PMC4794400 26422754

[87] Qi C Ding M Li S Zhou Q Li D Yu R. Sex-dependent modulation of immune development in mice by secretory IgA-coated Lactobacillus reuteri isolated from breast milk. J Dairy Sci (2021) 104(4):3863–75. doi: 10.3168/jds.2020-19437 33612242

[88] Li L Fang Z Liu X Hu W Lu W Lee YK. Lactobacillus reuteri attenuated allergic inflammation induced by HDM in the mouse and modulated gut microbes. PloS One (2020) 15(4):e0231865. doi: 10.1371/journal.pone.0231865 32315360 PMC7173794

[89] Gao G Li C Fan W Zhang M Li X Chen W. Brilliant glycans and glycosylation: Seq and ye shall find. Int J Biol macromolecules. (2021) 189:279–91. doi: 10.1016/j.ijbiomac.2021.08.054 34389387

[90] Shade KC Conroy ME Washburn N Kitaoka M Huynh DJ Laprise E. Sialylation of immunoglobulin E is a determinant of allergic pathogenicity. Nature (2020) 582(7811):265–70. doi: 10.1038/s41586-020-2311-z PMC7386252 32499653

[91] Fyhrquist N Muirhead G Prast-Nielsen S Jeanmougin M Olah P Skoog T. Microbe-host interplay in atopic dermatitis and psoriasis. Nat Commun (2019) 10(1):4703. doi: 10.1038/s41467-019-12253-y 31619666 PMC6795799

[92] Lipsky ZW Patsy M Marques CNH German GK. Mechanisms and implications of bacterial invasion across the human skin barrier. Microbiol Spectr. (2022) 10(3):e0274421. doi: 10.1128/spectrum.02744-21 35532353 PMC9241919

[93] Mizutani Y Takagi N Nagata H Inoue S. Interferon-gamma downregulates tight junction function, which is rescued by interleukin-17A. Exp Dermatol (2021) 30(12):1754–63. doi: 10.1111/exd.14425 PMC9290956 34197663

[94] Margolis DJ. Atopic dermatitis: filaggrin and skin barrier dysfunction. Br J Dermatol (2022) 186(3):396. doi: 10.1111/bjd.20946 35128630

[95] Dainichi T Kitoh A Otsuka A Nakajima S Nomura T Kaplan DH. The epithelial immune microenvironment (EIME) in atopic dermatitis and psoriasis. Nat Immunol (2018) 19(12):1286–98. doi: 10.1038/s41590-018-0256-2 30446754

[96] Akdis CA. Does the epithelial barrier hypothesis explain the increase in allergy, autoimmunity and other chronic conditions? Nat Rev Immunol (2021) 21(11):739–51. doi: 10.1038/s41577-021-00538-7 33846604

[97] Masiuk H Wcislek A Jursa-Kulesza J. Determination of nasal carriage and skin colonization, antimicrobial susceptibility and genetic relatedness of Staphylococcus aureus isolated from patients with atopic dermatitis in Szczecin, Poland. BMC Infect Dis (2021) 21(1):701. doi: 10.1186/s12879-021-06382-3 34294061 PMC8299601

[98] Luu LA Flowers RH Gao Y Wu M Gasperino S Kellams AL. Apple cider vinegar soaks do not alter the skin bacterial microbiome in atopic dermatitis. PloS One (2021) 16(6):e0252272. doi: 10.1371/journal.pone.0252272 34077434 PMC8172074

[99] Bjerre RD Holm JB Palleja A Solberg J Skov L Johansen JD. Skin dysbiosis in the microbiome in atopic dermatitis is site-specific and involves bacteria, fungus and virus. BMC Microbiol (2021) 21(1):256. doi: 10.1186/s12866-021-02302-2 34551705 PMC8459459

[100] Towell AM Feuillie C Vitry P Da Costa TM Mathelie-Guinlet M Kezic S. Staphylococcus aureus binds to the N-terminal region of corneodesmosin to adhere to the stratum corneum in atopic dermatitis. Proc Natl Acad Sci U.S.A. (2021) 118(1):e2014444118. doi: 10.1073/pnas.2014444118 33361150 PMC7817190

[101] Sung M Choi Y Park H Huh CS. Gut microbiome characteristics in mothers and infants according to the presence of atopic dermatitis. BioMed Res Int (2022) 2022:8145462. doi: 10.1155/2022/8145462 35502335 PMC9056221

[102] Jiang W Ni B Liu Z Liu X Xie W Wu IXY. The role of probiotics in the prevention and treatment of atopic dermatitis in children: an updated systematic review and meta-analysis of randomized controlled trials. Paediatr Drugs (2020) 22(5):535–49. doi: 10.1007/s40272-020-00410-6 32748341

[103] Navarro-Lopez V Ramirez-Bosca A Ramon-Vidal D Ruzafa-Costas B Genoves-Martinez S Chenoll-Cuadros E. Effect of oral administration of a mixture of probiotic strains on SCORAD index and use of topical steroids in young patients with moderate atopic dermatitis: A randomized clinical trial. JAMA Dermatol (2018) 154(1):37–43. doi: 10.1001/jamadermatol.2017.3647 29117309 PMC5833582

[104] Kim MJ Kim JY Kang M Won MH Hong SH Her Y. Reduced fecal calprotectin and inflammation in a murine model of atopic dermatitis following probiotic treatment. Int J Mol Sci (2020) 21(11):3968. doi: 10.3390/ijms21113968 32486523 PMC7312066

[105] Yoon W Park SH Lee JS Byeon JH Kim SH Lim J. Probiotic mixture reduces gut inflammation and microbial dysbiosis in children with atopic dermatitis. Australas J Dermatol (2021) 62(3):e386–e92. doi: 10.1111/ajd.13644 34110005

[106] Miniello VL Brunetti L Tesse R Natile M Armenio L Francavilla R. Lactobacillus reuteri modulates cytokines production in exhaled breath condensate of children with atopic dermatitis. J Pediatr Gastroenterol Nutr (2010) 50(5):573–6. doi: 10.1097/MPG.0b013e3181bb343f 20639717

[107] Zhou J Xu G Li X Tu H Li H Chang H. Limosilactobacillus reuteri FN041 prevents atopic dermatitis in pup mice by remodeling the ileal microbiota and regulating gene expression in Peyer's patches after vertical transmission. Front Nutr (2022) 9:987400. doi: 10.3389/fnut.2022.987400 36245510 PMC9554658

[108] Fang Z Pan T Wang H Zhu J Zhang H Zhao J. Limosilactobacillus reuteri Attenuates Atopic Dermatitis via Changes in Gut Bacteria and Indole Derivatives from Tryptophan Metabolism. Int J Mol Sci (2022) 23(14):7735. doi: 10.3390/ijms23147735 35887083 PMC9320942

[109] Forsythe P Inman MD Bienenstock J. Oral treatment with live Lactobacillus reuteri inhibits the allergic airway response in mice. Am J Respir Crit Care Med (2007) 175(6):561–9. doi: 10.1164/rccm.200606-821OC 17204726

[110] Moura JCV Moura ICG Gaspar GR Mendes GMS Faria BAV Jentzsch NS. The use of probiotics as a supplementary therapy in the treatment of patients with asthma: a pilot study and implications. Clinics (Sao Paulo). (2019) 74:e950. doi: 10.6061/clinics/2019/e950 31411278 PMC6683305

[111] Satia I Cusack R Stevens C Schlatman A Wattie J Mian F. Limosilactobacillus reuteri DSM-17938 for preventing cough in adults with mild allergic asthma: A double-blind randomized placebo-controlled cross-over study. Clin Exp Allergy (2021) 51(9):1133–43. doi: 10.1111/cea.13976 34192396

[112] Li L Fang Z Lee YK Zhao J Zhang H Peng H. Efficacy and safety of lactobacillus reuteri CCFM1040 in allergic rhinitis and asthma: A randomized, placebo-controlled trial. Front Nutr (2022) 9:862934. doi: 10.3389/fnut.2022.862934 35464005 PMC9022948

[113] Oncel MY Sari FN Arayici S Guzoglu N Erdeve O Uras N. Lactobacillus Reuteri for the prevention of necrotising enterocolitis in very low birthweight infants: a randomised controlled trial. Arch Dis Child Fetal Neonatal Ed (2014) 99(2):F110–5. doi: 10.1136/archdischild-2013-304745 24309022

[114] Liu Y Fatheree NY Mangalat N Rhoads JM. Lactobacillus reuteri strains reduce incidence and severity of experimental necrotizing enterocolitis via modulation of TLR4 and NF-kappaB signaling in the intestine. Am J Physiol Gastrointest Liver Physiol (2012) 302(6):G608–17. doi: 10.1152/ajpgi.00266.2011 PMC3311308 22207578

[115] Vadell AKE Barebring L Hulander E Gjertsson I Lindqvist HM Winkvist A. Anti-inflammatory Diet In Rheumatoid Arthritis (ADIRA)-a randomized, controlled crossover trial indicating effects on disease activity. Am J Clin Nutr (2020) 111(6):1203–13. doi: 10.1093/ajcn/nqaa019 PMC7266686 32055820

[116] Yeh YL Lu MC Tsai BC Tzang BS Cheng SM Zhang X. Heat-killed lactobacillus reuteri GMNL-263 inhibits systemic lupus erythematosus-induced cardiomyopathy in NZB/W F1 mice. Probiotics Antimicrob Proteins. (2021) 13(1):51–9. doi: 10.1007/s12602-020-09668-1 32514746

[117] Hsu TC Huang CY Liu CH Hsu KC Chen YH Tzang BS. Lactobacillus paracasei GMNL-32, Lactobacillus reuteri GMNL-89 and L. reuteri GMNL-263 ameliorate hepatic injuries in lupus-prone mice. Br J Nutr (2017) 117(8):1066–74. doi: 10.1017/S0007114517001039 28502277

[118] Fan Z Yang B Ross RP Stanton C Zhao J Zhang H. The prophylactic effects of different Lactobacilli on collagen-induced arthritis in rats. Food Funct (2020) 11(4):3681–94. doi: 10.1039/C9FO02556A 32301444

[119] Rothhammer V Mascanfroni ID Bunse L Takenaka MC Kenison JE Mayo L. Type I interferons and microbial metabolites of tryptophan modulate astrocyte activity and central nervous system inflammation via the aryl hydrocarbon receptor. Nat Med (2016) 22(6):586–97. doi: 10.1038/nm.4106 PMC4899206 27158906

[120] He B Hoang TK Tian X Taylor CM Blanchard E Luo M. Lactobacillus reuteri reduces the severity of experimental autoimmune encephalomyelitis in mice by modulating gut microbiota. Front Immunol (2019) 10:385. doi: 10.3389/fimmu.2019.00385 30899262 PMC6416370

[121] Li H Zhang Z Zhang H Guo Y Yao Z. Update on the pathogenesis and therapy of atopic dermatitis. Clin Rev Allergy Immunol (2021) 61(3):324–38. doi: 10.1007/s12016-021-08880-3 34338977

[122] Meng J Li Y Fischer MJM Steinhoff M Chen W Wang J. Th2 modulation of transient receptor potential channels: an unmet therapeutic intervention for atopic dermatitis. Front Immunol (2021) 12:696784. doi: 10.3389/fimmu.2021.696784 34276687 PMC8278285

[123] Protti MP De Monte L. Thymic stromal lymphopoietin and cancer: th2-dependent and -independent mechanisms. Front Immunol (2020) 11:2088. doi: 10.3389/fimmu.2020.02088 33042121 PMC7524868

[124] He R Geha RS. Thymic stromal lymphopoietin. Ann N Y Acad Sci (2010) 1183:13–24. doi: 10.1111/j.1749-6632.2009.05128.x 20146705 PMC2895428

[125] Pernomian L Duarte-Silva M de Barros Cardoso CR. The aryl hydrocarbon receptor (AHR) as a potential target for the control of intestinal inflammation: insights from an immune and bacteria sensor receptor. Clin Rev Allergy Immunol (2020) 59(3):382–90. doi: 10.1007/s12016-020-08789-3 32279195

[126] Alexeev EE Lanis JM Kao DJ Campbell EL Kelly CJ Battista KD. Microbiota-derived indole metabolites promote human and murine intestinal homeostasis through regulation of Interleukin-10 receptor. Am J Pathol (2018) 188(5):1183–94. doi: 10.1016/j.ajpath.2018.01.011 PMC5906738 29454749

[127] AMalia N Orchard D Francis KL King E. Systematic review and meta-analysis on the use of probiotic supplementation in pregnant mother, breastfeeding mother and infant for the prevention of atopic dermatitis in children. Australas J Dermatol (2020) 61(2):e158–e73. doi: 10.1111/ajd.13186 31721162

[128] Lunjani N Hlela C O'Mahony L. Microbiome and skin biology. Curr Opin Allergy Clin Immunol (2019) 19(4):328–33. doi: 10.1097/ACI.0000000000000542 31107258

[129] Shi B Li W Dong H Xu M Hao Y Gao P. Distribution of inflammatory phenotypes among patients with asthma in Jilin Province, China: a cross-sectional study. BMC Pulm Med (2021) 21(1):364. doi: 10.1186/s12890-021-01722-0 34772390 PMC8590234

[130] Lambrecht BN Hammad H Fahy JV. The cytokines of asthma. Immunity (2019) 50(4):975–91. doi: 10.1016/j.immuni.2019.03.018 30995510

[131] Chen M Shepard K 2nd Yang M Raut P Pazwash H Holweg CTJ. Overlap of allergic, eosinophilic and type 2 inflammatory subtypes in moderate-to-severe asthma. Clin Exp Allergy (2021) 51(4):546–55. doi: 10.1111/cea.13790 PMC8048421 33217063

[132] McCauley KE Flynn K Calatroni A DiMassa V LaMere B Fadrosh DW. Seasonal airway microbiome and transcriptome interactions promote childhood asthma exacerbations. J Allergy Clin Immunol (2022) 150(1):204–13. doi: 10.1016/j.jaci.2022.01.020 35149044

[133] Ahluwalia TS Eliasen AU Sevelsted A Pedersen CT Stokholm J Chawes B. FUT2-ABO epistasis increases the risk of early childhood asthma and Streptococcus pneumoniae respiratory illnesses. Nat Commun (2020) 11(1):6398. doi: 10.1038/s41467-020-19814-6 33328473 PMC7744576

[134] Diver S Richardson M Haldar K Ghebre MA Ramsheh MY Bafadhel M. Sputum microbiomic clustering in asthma and chronic obstructive pulmonary disease reveals a Haemophilus-predominant subgroup. Allergy (2020) 75(4):808–17. doi: 10.1111/all.14058 PMC7217013 31556120

[135] Zhou Y Jackson D Bacharier LB Mauger D Boushey H Castro M. The upper-airway microbiota and loss of asthma control among asthmatic children. Nat Commun (2019) 10(1):5714. doi: 10.1038/s41467-019-13698-x 31844063 PMC6915697

[136] Huang C Ni Y Du W Shi G. Effect of inhaled corticosteroids on microbiome and microbial correlations in asthma over a 9-month period. Clin Transl Sci (2022) 15(7):1723–36. doi: 10.1111/cts.13288 PMC9283747 35514165

[137] Budden KF Gellatly SL Wood DL Cooper MA Morrison M Hugenholtz P. Emerging pathogenic links between microbiota and the gut-lung axis. Nat Rev Microbiol (2017) 15(1):55–63. doi: 10.1038/nrmicro.2016.142 27694885

[138] Dumas A Bernard L Poquet Y Lugo-Villarino G Neyrolles O. The role of the lung microbiota and the gut-lung axis in respiratory infectious diseases. Cell Microbiol (2018) 20(12):e12966. doi: 10.1111/cmi.12966 30329198

[139] Marsland BJ Trompette A Gollwitzer ES. The gut-lung axis in respiratory disease. Ann Am Thorac Soc (2015) 12 Suppl 2:S150–6. doi: 10.1513/AnnalsATS.201503-133AW 26595731

[140] Frati F Salvatori C Incorvaia C Bellucci A Di Cara G Marcucci F. The role of the microbiome in asthma: the gut(-)Lung axis. Int J Mol Sci (2018) 20(1):123. doi: 10.3390/ijms20010123 30598019 PMC6337651

[141] Durack J Kimes NE Lin DL Rauch M McKean M McCauley K. Delayed gut microbiota development in high-risk for asthma infants is temporarily modifiable by Lactobacillus supplementation. Nat Commun (2018) 9(1):707. doi: 10.1038/s41467-018-03157-4 29453431 PMC5816017

[142] Stokholm J Blaser MJ Thorsen J Rasmussen MA Waage J Vinding RK. Maturation of the gut microbiome and risk of asthma in childhood. Nat Commun (2018) 9(1):141. doi: 10.1038/s41467-017-02573-2 29321519 PMC5762761

[143] Krzych-Falta E Furmanczyk K Tomaszewska A Olejniczak D Samolinski B Samolinska-Zawisza U. Probiotics: Myths or facts about their role in allergy prevention. Adv Clin Exp Med (2018) 27(1):119–24. doi: 10.17219/acem/65476 29521052

[144] Liu A Ma T Xu N Jin H Zhao F Kwok LY. Adjunctive probiotics alleviates asthmatic symptoms via modulating the gut microbiome and serum metabolome. Microbiol Spectr. (2021) 9(2):e0085921. doi: 10.1128/Spectrum.00859-21 34612663 PMC8510161

[145] Chen JC Tsai CC Hsieh CC Lan A Huang CC Leu SF. Multispecies probiotics combination prevents ovalbumin-induced airway hyperreactivity in mice. Allergol Immunopathol (Madr). (2018) 46(4):354–60. doi: 10.1016/j.aller.2018.02.001 29739682

[146] Cervantes-Garcia D Jimenez M Rivas-Santiago CE Gallegos-Alcala P Hernandez-Mercado A Santoyo-Payan LS. Lactococcus lactis NZ9000 Prevents Asthmatic Airway Inflammation and Remodelling in Rats through the Improvement of Intestinal Barrier Function and Systemic TGF-beta Production. Int Arch Allergy Immunol (2021) 182(4):277–91. doi: 10.1159/000511146 33147596

[147] Jamalkandi SA Ahmadi A Ahrari I Salimian J Karimi M Ghanei M. Oral and nasal probiotic administration for the prevention and alleviation of allergic diseases, asthma and chronic obstructive pulmonary disease. Nutr Res Rev (2021) 34(1):1–16. doi: 10.1017/S0954422420000116 32281536

[148] Bellodas Sanchez J Kadrofske M. Necrotizing enterocolitis. Neurogastroenterol Motil. (2019) 31(3):e13569. doi: 10.1111/nmo.13569 30793842

[149] Imren C Vlug LE de Koning BAE Diertens T Snel HE Suurland J. Necrotizing enterocolitis in a dutch cohort of very preterm infants: prevalence, mortality, and long-term outcomes. Eur J Pediatr Surg (2022) 32(1):111–9. doi: 10.1055/s-0041-1741544 35008115

[150] Jia CH Feng ZS Lin XJ Cui QL Han SS Jin Y. Short term outcomes of extremely low birth weight infants from a multicenter cohort study in Guangdong of China. Sci Rep (2022) 12(1):11119. doi: 10.1038/s41598-022-14432-2 35778441 PMC9249781

[151] Nino DF Sodhi CP Hackam DJ. Necrotizing enterocolitis: new insights into pathogenesis and mechanisms. Nat Rev Gastroenterol Hepatol (2016) 13(10):590–600. doi: 10.1038/nrgastro.2016.119 27534694 PMC5124124

[152] Chen WY Lo YC Huang PH Chen YX Tsao PC Lee YS. Increased antibiotic exposure in early life is associated with adverse outcomes in very low birth weight infants. J Chin Med Assoc (2022) 85(9):939–43. doi: 10.1097/JCMA.0000000000000749 PMC12755642 35648148

[153] Pammi M Cope J Tarr PI Warner BB Morrow AL Mai V. Intestinal dysbiosis in preterm infants preceding necrotizing enterocolitis: a systematic review and meta-analysis. Microbiome (2017) 5(1):31. doi: 10.1186/s40168-017-0248-8 28274256 PMC5343300

[154] He Y Du W Xiao S Zeng B She X Liu D. Colonization of fecal microbiota from patients with neonatal necrotizing enterocolitis exacerbates intestinal injury in germfree mice subjected to necrotizing enterocolitis-induction protocol via alterations in butyrate and regulatory T cells. J Transl Med (2021) 19(1):510. doi: 10.1186/s12967-021-03109-5 34922582 PMC8684079

[155] Schonherr-Hellec S Klein GL Delannoy J Ferraris L Roze JC Butel MJ. Clostridial strain-specific characteristics associated with necrotizing enterocolitis. Appl Environ Microbiol (2018) 84(7):e02428–17. doi: 10.1128/AEM.02428-17 PMC5861827 29352082

[156] Tarracchini C Milani C Longhi G Fontana F Mancabelli L Pintus R. Unraveling the microbiome of necrotizing enterocolitis: insights in novel microbial and metabolomic biomarkers. Microbiol Spectr. (2021) 9(2):e0117621. doi: 10.1128/Spectrum.01176-21 34704805 PMC8549755

[157] Olm MR Bhattacharya N Crits-Christoph A Firek BA Baker R Song YS. Necrotizing enterocolitis is preceded by increased gut bacterial replication, Klebsiella, and fimbriae-encoding bacteria. Sci Adv (2019) 5(12):eaax5727. doi: 10.1126/sciadv.aax5727 31844663 PMC6905865

[158] Paveglio S Ledala N Rezaul K Lin Q Zhou Y Provatas AA. Cytotoxin-producing Klebsiella oxytoca in the preterm gut and its association with necrotizing enterocolitis. Emerg Microbes Infect (2020) 9(1):1321–9. doi: 10.1080/22221751.2020.1773743 PMC7473113 32525754

[159] Itani T Ayoub Moubareck C Melki I Rousseau C Mangin I Butel MJ. Preterm infants with necrotising enterocolitis demonstrate an unbalanced gut microbiota. Acta Paediatr (2018) 107(1):40–7. doi: 10.1111/apa.14078 28921627

[160] Stafford IA Rodrigue E Berra A Adams W Heard AJ Hagan JL. The strong correlation between neonatal early-onset Group B Streptococcal disease and necrotizing enterocolitis. Eur J Obstet Gynecol Reprod Biol (2018) 223:93–7. doi: 10.1016/j.ejogrb.2018.02.024 29501938

[161] Brunse A Deng L Pan X Hui Y Castro-Mejia JL Kot W. Fecal filtrate transplantation protects against necrotizing enterocolitis. ISME J (2022) 16(3):686–94. doi: 10.1038/s41396-021-01107-5 PMC8857206 34552194

[162] Morgan RL Preidis GA Kashyap PC Weizman AV Sadeghirad B McMaster Probiotic P. Probiotics reduce mortality and morbidity in preterm, low-birth-weight infants: A systematic review and network meta-analysis of randomized trials. Gastroenterology (2020) 159(2):467–80. doi: 10.1053/j.gastro.2020.05.096 PMC8014956 32592699

[163] Sowden M van Weissenbruch MM Bulabula ANH van Wyk L Twisk J van Niekerk E. Effect of a multi-strain probiotic on the incidence and severity of necrotizing enterocolitis and feeding intolerances in preterm neonates. Nutrients (2022) 14(16):3305. doi: 10.3390/nu14163305 36014810 PMC9415863

[164] Delaplain PT Bell BA Wang J Isani M Zhang E Gayer CP. Effects of artificially introduced Enterococcus faecalis strains in experimental necrotizing enterocolitis. PloS One (2019) 14(11):e0216762. doi: 10.1371/journal.pone.0216762 31675374 PMC6824573

[165] Hui Y Smith B Mortensen MS Krych L Sorensen SJ Greisen G. The effect of early probiotic exposure on the preterm infant gut microbiome development. Gut Microbes (2021) 13(1):1951113. doi: 10.1080/19490976.2021.1951113 34264803 PMC8284123

[166] Yu R Jiang S Tao Y Li P Yin J Zhou Q. Inhibition of HMGB1 improves necrotizing enterocolitis by inhibiting NLRP3 via TLR4 and NF-kappaB signaling pathways. J Cell Physiol (2019) 234(8):13431–8. doi: 10.1002/jcp.28022 PMC13483282 30618088

[167] Sun Q Ji YC Wang ZL She X He Y Ai Q. Sodium butyrate alleviates intestinal inflammation in mice with necrotizing enterocolitis. Mediators Inflamm (2021) 2021:6259381. doi: 10.1155/2021/6259381 34675753 PMC8526205

[168] Le Mandat Schultz A Bonnard A Barreau F Aigrain Y Pierre-Louis C Berrebi D. Expression of TLR-2, TLR-4, NOD2 and pNF-kappaB in a neonatal rat model of necrotizing enterocolitis. PloS One (2007) 2(10):e1102. doi: 10.1371/journal.pone.0001102 17971865 PMC2040507

[169] Hunter C Dimaguila MA Gal P Wimmer JE Jr. Ransom JL Carlos RQ. Effect of routine probiotic, Lactobacillus reuteri DSM 17938, use on rates of necrotizing enterocolitis in neonates with birthweight < 1000 grams: a sequential analysis. BMC Pediatr (2012) 12:142. doi: 10.1186/1471-2431-12-142 22947597 PMC3472183

[170] Silverman GJ Deng J Azzouz DF. Sex-dependent Lupus Blautia (Ruminococcus) gnavus strain induction of zonulin-mediated intestinal permeability and autoimmunity. Front Immunol (2022) 13:897971. doi: 10.3389/fimmu.2022.897971 36032126 PMC9405438

[171] Ma Y Guo R Sun Y Li X He L Li Z. Lupus gut microbiota transplants cause autoimmunity and inflammation. Clin Immunol (2021) 233:108892. doi: 10.1016/j.clim.2021.108892 34813937

[172] Xiang K Wang P Xu Z Hu YQ He YS Chen Y. Causal effects of gut microbiome on systemic lupus erythematosus: A two-sample mendelian randomization study. Front Immunol (2021) 12:667097. doi: 10.3389/fimmu.2021.667097 34557183 PMC8453215

[173] Liu F Ren T Li X Zhai Q Xu X Zhang N. Distinct microbiomes of gut and saliva in patients with systemic lupus erythematous and clinical associations. Front Immunol (2021) 12:626217. doi: 10.3389/fimmu.2021.626217 34276643 PMC8281017

[174] He J Chan T Hong X Zheng F Zhu C Yin L. Microbiome and metabolome analyses reveal the disruption of lipid metabolism in systemic lupus erythematosus. Front Immunol (2020) 11:1703. doi: 10.3389/fimmu.2020.01703 32849599 PMC7411142

[175] Tomofuji Y Maeda Y Oguro-Igashira E Kishikawa T Yamamoto K Sonehara K. Metagenome-wide association study revealed disease-specific landscape of the gut microbiome of systemic lupus erythematosus in Japanese. Ann Rheum Dis (2021) 80(12):1575–83. doi: 10.1136/annrheumdis-2021-220687 PMC8600607 34426398

[176] Chen BD Jia XM Xu JY Zhao LD Ji JY Wu BX. An autoimmunogenic and proinflammatory profile defined by the gut microbiota of patients with untreated systemic lupus erythematosus. Arthritis Rheumatol (2021) 73(2):232–43. doi: 10.1002/art.41511 33124780

[177] Shirakashi M Maruya M Hirota K Tsuruyama T Matsuo T Watanabe R. Effect of impaired T cell receptor signaling on the gut microbiota in a mouse model of systemic autoimmunity. Arthritis Rheumatol (2022) 74(4):641–53. doi: 10.1002/art.42016 34725966

[178] Azzouz D Omarbekova A Heguy A Schwudke D Gisch N Rovin BH. Lupus nephritis is linked to disease-activity associated expansions and immunity to a gut commensal. Ann Rheum Dis (2019) 78(7):947–56. doi: 10.1136/annrheumdis-2018-214856 PMC6585303 30782585

[179] Li Y Wang HF Li X Li HX Zhang Q Zhou HW. Disordered intestinal microbes are associated with the activity of Systemic Lupus Erythematosus. Clin Sci (Lond). (2019) 133(7):821–38. doi: 10.1042/CS20180841 30872359

[180] Ma Y Xu X Li M Cai J Wei Q Niu H. Gut microbiota promote the inflammatory response in the pathogenesis of systemic lupus erythematosus. Mol Med (2019) 25(1):35. doi: 10.1186/s10020-019-0102-5 31370803 PMC6676588

[181] Tampe D Hakroush S Tampe B. Dissecting signalling pathways associated with intrarenal synthesis of complement components in lupus nephritis. RMD Open (2022) 8(2):e00251. doi: 10.1136/rmdopen-2022-002517 PMC9345095 35906025

[182] Pucci G Vaudo G. Cardiac and vascular damage in systemic erythematosus lupus. Is Dis activity mediator? Eur J Intern Med (2020) 73:23–4. doi: 10.1016/j.ejim.2020.01.013 32001097

[183] Mauro D Nerviani A. Endothelial dysfunction in systemic lupus erythematosus: pathogenesis, assessment and therapeutic opportunities. Rev Recent Clin Trials. (2018) 13(3):192–8. doi: 10.2174/1574887113666180314091831 29542419

[184] de la Visitacion N Robles-Vera I Toral M O'Valle F Moleon J Gomez-Guzman M. Lactobacillus fermentum CECT5716 prevents renal damage in the NZBWF1 mouse model of systemic lupus erythematosus. Food Funct (2020) 11(6):5266–74. doi: 10.1039/D0FO00578A 32458936

[185] Hu WS Rajendran P Tzang BS Yeh YL Shen CY Chen RJ. Lactobacillus paracasei GMNL-32 exerts a therapeutic effect on cardiac abnorMalities in NZB/W F1 mice. PloS One (2017) 12(9):e0185098. doi: 10.1371/journal.pone.0185098 28934296 PMC5608316

[186] de la Visitacion N Robles-Vera I Moleon-Moya J Sanchez M Jimenez R Gomez-Guzman M. Probiotics prevent hypertension in a murine model of systemic lupus erythematosus induced by toll-like receptor 7 activation. Nutrients (2021) 13(8):2669. doi: 10.3390/nu13082669 34444829 PMC8399640

[187] Zhang Y Murugesan P Huang K Cai H. NADPH oxidases and oxidase crosstalk in cardiovascular diseases: novel therapeutic targets. Nat Rev Cardiol (2020) 17(3):170–94. doi: 10.1038/s41569-019-0260-8 PMC7880919 31591535

[188] Tschudi MR Mesaros S Luscher TF Malinski T. Direct in *situ* measurement of nitric oxide in mesenteric resistance arteries. Increased decomposition by superoxide in hypertension. Hypertension (1996) 27(1):32–5. doi: 10.1161/01.HYP.27.1.32 8591884

[189] Mardani F Mahmoudi M Esmaeili SA Khorasani S Tabasi N Rastin M. *In vivo* study: Th1-Th17 reduction in pristane-induced systemic lupus erythematosus mice after treatment with tolerogenic Lactobacillus probiotics. J Cell Physiol (2018) 234(1):642–9. doi: 10.1002/jcp.26819 30078223

[190] Wang X Xia Y. Anti-double stranded DNA antibodies: origin, pathogenicity, and targeted therapies. Front Immunol (2019) 10:1667. doi: 10.3389/fimmu.2019.01667 31379858 PMC6650533

[191] Wang Y Xiao S Xia Y Wang H. The therapeutic strategies for SLE by targeting anti-dsDNA antibodies. Clin Rev Allergy Immunol (2022) 63(2):152–65. doi: 10.1007/s12016-021-08898-7 PMC9464114 34542806

[192] Pisetsky DS Lipsky PE. New insights into the role of antinuclear antibodies in systemic lupus erythematosus. Nat Rev Rheumatol (2020) 16(10):565–79. doi: 10.1038/s41584-020-0480-7 PMC8456518 32884126

[193] Li PH Wong WH Lee TL Lau CS Chan TM Leung AM. Relationship between autoantibody clustering and clinical subsets in SLE: cluster and association analyses in Hong Kong Chinese. Rheumatol (Oxford). (2013) 52(2):337–45. doi: 10.1093/rheumatology/kes261 23038697

[194] Talaat RM Mohamed SF Bassyouni IH Raouf AA. Th1/Th2/Th17/Treg cytokine imbalance in systemic lupus erythematosus (SLE) patients: Correlation with disease activity. Cytokine (2015) 72(2):146–53. doi: 10.1016/j.cyto.2014.12.027 25647269

[195] Alvarez-Rodriguez L Martinez-Taboada V Calvo-Alen J Beares I Villa I Lopez-Hoyos M. Altered Th17/treg ratio in peripheral blood of systemic lupus erythematosus but not primary antiphospholipid syndrome. Front Immunol (2019) 10:391. doi: 10.3389/fimmu.2019.00391 30894863 PMC6414457

[196] Yuliasih Y Rahmawati LD Putri RM. Th17/treg ratio and disease activity in systemic lupus erythematosus. Caspian J Intern Med (2019) 10(1):65–72. doi: 10.22088/cjim.10.1.65 30858943 PMC6386323

[197] Li D Pan Y Xia X Liang J Liu F Dou H. Bacteroides fragilis alleviates the symptoms of lupus nephritis *via* regulating CD1d and CD86 expressions in B cells. Eur J Pharmacol (2020) 884:173421. doi: 10.1016/j.ejphar.2020.173421 32721450

[198] Kim DS Park Y Choi JW Park SH Cho ML Kwok SK. Lactobacillus acidophilus Supplementation Exerts a Synergistic Effect on Tacrolimus Efficacy by Modulating Th17/Treg Balance in Lupus-Prone Mice *via* the SIGNR3 Pathway. Front Immunol (2021) 12:696074. doi: 10.3389/fimmu.2021.696074 34956169 PMC8704231

[199] Zhou T Lin S Yang S Lin W. Efficacy and safety of tacrolimus in induction therapy of patients with lupus nephritis. Drug Des Devel Ther (2019) 13:857–69. doi: 10.2147/DDDT.S189156 PMC6420100 30880918

[200] Suzuki K Kameda H Amano K Nagasawa H Takei H Nishi E. Single center prospective study of tacrolimus efficacy and safety in the treatment of various manifestations in systemic lupus erythematosus. Rheumatol Int (2011) 31(6):757–63. doi: 10.1007/s00296-010-1366-9 20169348

[201] Tani C Elefante E Martin-Cascon M Belhocine M Lavilla Olleros C Vagelli R. Tacrolimus in non-Asian patients with SLE: a real-life experience from three European centres. Lupus Sci Med (2018) 5(1):e000274. doi: 10.1136/lupus-2018-000274 30538815 PMC6257376

[202] Park JS Kim SM Hwang SH Choi SY Kwon JY Kwok SK. Combinatory treatment using tacrolimus and a STAT3 inhibitor regulate Treg cells and plasma cells. Int J Immunopathol Pharmacol (2018) 32:2058738418778724. doi: 10.1177/2058738418778724 29873267 PMC5992791

[203] Mueller AL Payandeh Z Mohammadkhani N Mubarak SMH Zakeri A Alagheband Bahrami A. Recent advances in understanding the pathogenesis of rheumatoid arthritis: new treatment strategies. Cells (2021) 10(11):3017. doi: 10.3390/cells10113017 34831240 PMC8616543

[204] Qiu J Wu B Goodman SB Berry GJ Goronzy JJ Weyand CM. Metabolic control of autoimmunity and tissue inflammation in rheumatoid arthritis. Front Immunol (2021) 12:652771. doi: 10.3389/fimmu.2021.652771 33868292 PMC8050350

[205] Zhou N Zou F Cheng X Huang Y Zou H Niu Q. Porphyromonas gingivalis induces periodontitis, causes immune imbalance, and promotes rheumatoid arthritis. J Leukoc Biol (2021) 110(3):461–73. doi: 10.1002/JLB.3MA0121-045R 34057740

[206] Cheng Z Do T Mankia K Meade J Hunt L Clerehugh V. Dysbiosis in the oral microbiomes of anti-CCP positive individuals at risk of developing rheumatoid arthritis. Ann Rheum Dis (2021) 80(2):162–8. doi: 10.1136/annrheumdis-2020-216972 33004333

[207] Kroese JM Brandt BW Buijs MJ Crielaard W Lobbezoo F Loos BG. Differences in the oral microbiome in patients with early rheumatoid arthritis and individuals at risk of rheumatoid arthritis compared to healthy individuals. Arthritis Rheumatol (2021) 73(11):1986–93. doi: 10.1002/art.41780 PMC8596438 33949151

[208] Kishikawa T Maeda Y Nii T Motooka D Matsumoto Y Matsushita M. Metagenome-wide association study of gut microbiome revealed novel aetiology of rheumatoid arthritis in the Japanese population. Ann Rheum Dis (2020) 79(1):103–11. doi: 10.1136/annrheumdis-2019-215743 PMC6937407 31699813

[209] Jubair WK Hendrickson JD Severs EL Schulz HM Adhikari S Ir D. Modulation of inflammatory arthritis in mice by gut microbiota through mucosal inflammation and autoantibody generation. Arthritis Rheumatol (2018) 70(8):1220–33. doi: 10.1002/art.40490 PMC6105374 29534332

[210] Kitamura K Shionoya H Suzuki S Fukai R Uda S Abe C. Oral and intestinal bacterial substances associated with disease activities in patients with rheumatoid arthritis: A cross-sectional clinical study. J Immunol Res (2022) 2022:6839356. doi: 10.1155/2022/6839356 35224112 PMC8881124

[211] Cannarella LAT Mari NL Alcantara CC Iryioda TMV Costa NT Oliveira SR. Mixture of probiotics reduces inflammatory biomarkers and improves the oxidative/nitrosative profile in people with rheumatoid arthritis. Nutrition (2021) 89:111282. doi: 10.1016/j.nut.2021.111282 34111674

[212] Dourado E Ferro M Sousa Guerreiro C Fonseca JE. Diet as a modulator of intestinal microbiota in rheumatoid arthritis. Nutrients (2020) 12(11):3504. doi: 10.3390/nu12113504 33202579 PMC7696404

[213] Gioia C Lucchino B Tarsitano MG Iannuccelli C Di Franco M. Dietary habits and nutrition in rheumatoid arthritis: can diet influence disease development and clinical manifestations? Nutrients (2020) 12(5):1456. doi: 10.3390/nu12051456 32443535 PMC7284442

[214] Hulander E Barebring L Turesson Wadell A Gjertsson I Calder PC Winkvist A. Diet intervention improves cardiovascular profile in patients with rheumatoid arthritis: results from the randomized controlled cross-over trial ADIRA. Nutr J (2021) 20(1):9. doi: 10.1186/s12937-021-00663-y 33485336 PMC7827982

[215] Wang Y Zhu D Ortiz-Velez LC Perry JL Pennington MW Hyser JM. A bioengineered probiotic for the oral delivery of a peptide Kv1.3 channel blocker to treat rheumatoid arthritis. Proc Natl Acad Sci U.S.A. (2023) 120(2):e2211977120. doi: 10.1073/pnas.2211977120 36595694 PMC9926172

[216] Hemmer B Kerschensteiner M Korn T. Role of the innate and adaptive immune responses in the course of multiple sclerosis. Lancet Neurol (2015) 14(4):406–19. doi: 10.1016/S1474-4422(14)70305-9 25792099

[217] Rodriguez Murua S Farez MF Quintana FJ. The immune response in multiple sclerosis. Annu Rev Pathol (2022) 17:121–39. doi: 10.1146/annurev-pathol-052920-040318 34606377

[218] Elsayed NS Aston P Bayanagari VR Shukla SK. The gut microbiome molecular mimicry piece in the multiple sclerosis puzzle. Front Immunol (2022) 13:972160. doi: 10.3389/fimmu.2022.972160 36045671 PMC9420973

[219] Bianchimano P Britton GJ Wallach DS Smith EM Cox LM Liu S. Mining the microbiota to identify gut commensals modulating neuroinflammation in a mouse model of multiple sclerosis. Microbiome (2022) 10(1):174. doi: 10.1186/s40168-022-01364-2 36253847 PMC9575236

[220] Cox LM Maghzi AH Liu S Tankou SK Dhang FH Willocq V. Gut microbiome in progressive multiple sclerosis. Ann Neurol (2021) 89(6):1195–211. doi: 10.1002/ana.26084 PMC8132291 33876477

[221] Reynders T Devolder L Valles-Colomer M Van Remoortel A Joossens M De Keyser J. Gut microbiome variation is associated to Multiple Sclerosis phenotypic subtypes. Ann Clin Transl Neurol (2020) 7(4):406–19. doi: 10.1002/acn3.51004 PMC7187717 32162850

[222] Gandy KAO Zhang J Nagarkatti P Nagarkatti M. The role of gut microbiota in shaping the relapse-remitting and chronic-progressive forms of multiple sclerosis in mouse models. Sci Rep (2019) 9(1):6923. doi: 10.1038/s41598-019-43356-7 31061496 PMC6502871

[223] Horton MK McCauley K Fadrosh D Fujimura K Graves J Ness J. Gut microbiome is associated with multiple sclerosis activity in children. Ann Clin Transl Neurol (2021) 8(9):1867–83. doi: 10.1002/acn3.51441 PMC8419410 34409759

[224] Levi I Gurevich M Perlman G Magalashvili D Menascu S Bar N. Potential role of indolelactate and butyrate in multiple sclerosis revealed by integrated microbiome-metabolome analysis. Cell Rep Med (2021) 2(4):100246. doi: 10.1016/j.xcrm.2021.100246 33948576 PMC8080254

[225] Roager HM Licht TR. Microbial tryptophan catabolites in health and disease. Nat Commun (2018) 9(1):3294. doi: 10.1038/s41467-018-05470-4 30120222 PMC6098093

[226] Chyan YJ Poeggeler B Omar RA Chain DG Frangione B Ghiso J. Potent neuroprotective properties against the Alzheimer beta-amyloid by an endogenous melatonin-related indole structure, indole-3-propionic acid. J Biol Chem (1999) 274(31):21937–42. doi: 10.1074/jbc.274.31.21937 10419516

[227] Tankou SK Regev K Healy BC Cox LM Tjon E Kivisakk P. Investigation of probiotics in multiple sclerosis. Multiple sclerosis (Houndmills Basingstoke England). (2018) 24(1):58–63. doi: 10.1177/1352458517737390 29307299

[228] Rahimlou M Hosseini SA Majdinasab N Haghighizadeh MH Husain D. Effects of long-term administration of Multi-Strain Probiotic on circulating levels of BDNF, NGF, IL-6 and mental health in patients with multiple sclerosis: a randomized, double-blind, placebo-controlled trial. Nutr Neurosci (2022) 25(2):411–22. doi: 10.1080/1028415X.2020.1758887 32500827

[229] Tanis KQ Newton SS Duman RS. Targeting neurotrophic/growth factor expression and signaling for antidepressant drug development. CNS neurological Disord Drug targets. (2007) 6(2):151–60. doi: 10.2174/187152707780363276 17430152

[230] Montgomery TL Kunstner A Kennedy JJ Fang Q Asarian L Culp-Hill R. Interactions between host genetics and gut microbiota determine susceptibility to CNS autoimmunity. Proc Natl Acad Sci U S A. (2020) 117(44):27516–27. doi: 10.1073/pnas.2002817117 PMC7959502 33077601

